# Relative contributions of lifestyle factors to stage-specific progression and mortality in cardiovascular-kidney-metabolic syndrome: a prospective cohort study with multi-state models

**DOI:** 10.3389/fpubh.2026.1782700

**Published:** 2026-03-20

**Authors:** Qi Chen, Nian Duan, Shenrui Li, Fenghuixue Liu, Ping Yin

**Affiliations:** 1 Department of Epidemiology and Biostatistics, School of Public Health, Tongji Medical College, Huazhong University of Science and Technology, Wuhan, China; 2 Department of Day Surgery, The Central Hospital of Wuhan, Tongji Medical College, Huazhong University of Science and Technology, Wuhan, China

**Keywords:** cardiovascular disease, cardiovascular-kidney-metabolic syndrome, disease progression, lifestyle factors, multi-state models, UK biobank

## Abstract

**Background:**

Cardiovascular-kidney-metabolic (CKM) syndrome, a novel multisystem disorder, integrates interconnected risks across cardiovascular, renal, and metabolic systems. While lifestyle factors are known to influence each CKM component disease, their impact on the dynamic progression of CKM syndrome as a unified entity remains unclear, particularly across its longitudinal stages and clinical subtypes (4a/4b).

**Methods:**

The study included 308,657 United Kingdom Biobank participants in CKM stages 0–3 at baseline. Multi-state models were used to investigate the effects of composite and individual lifestyle scores (smoking, alcohol consumption, physical activity, diet, sleep duration, and sedentary time) on transitions to clinical stage and subsequent death. The relative contribution of each factor was quantified using quantile G-computation.

**Results:**

During a median follow-up of 13.71 years, 30,516 participants progressed to stage 4 and 22,280 deaths occurred. Each 1-point improvement in lifestyle score was associated with approximately a 15% lower risk of CKM progression. After refining stage 4 into subtypes, the strongest protective effect was observed in transition from baseline to stage 4b, with hazard ratios (HRs) of 0.57 [95% confidence interval (CI): 0.52–0.63] for the score 2–4 group and 0.35 (95% CI: 0.30–0.41) for the score 5–6 group, compared to score 0–1. Never smoking showed the greatest contribution overall (23.1–65.1%) across stage transitions, with the strongest risk reduction observed in the transition from stage 4a to death (HR = 0.65, 95% CI: 0.60–0.70).

**Conclusion:**

A healthy lifestyle was associated with slower longitudinal CKM syndrome progression. These findings support stage-specific lifestyle strategies and early intervention, emphasizing smoking cessation as a key target to delay CKM progression and reduce premature death.

## 1 Introduction

Growing evidence suggests that cardiovascular disease (CVD), chronic kidney disease (CKD), and metabolic diseases are closely interconnected. They share overlapping mechanisms and risk factors, and dysfunction in one system may accelerate damage in the others (1, 2). In 2023, the American Heart Association (AHA) introduced an integrative framework for the cardiovascular-kidney-metabolic (CKM) syndrome, offering a foundation for systemic understanding and precision prevention (3).

As a structured framework for stratifying risk and informing early intervention, CKM staging delineates progression from stage 0 (absence of risk factors) to stage 4 (clinical CVD, with or without renal dysfunction). The risk of death incrementally increases with advancing stages, with premature mortality primarily driven by clinical CVD at stage 4 (4, 5). Furthermore, a study in the US reported that approximately 90.9% of adults are in stages 0–3 of CKM syndrome (6). Therefore, studies targeting the CKM stages 0–3, which represent varying degrees of risk and subclinical damage before clinical CVD onset, are essential for slowing progression to stage 4 and reducing clinical CVD burden. Nevertheless, existing research has predominantly used cross-sectional methods, and comprehensive assessments of CKM long-term progression and mortality risk remain limited (7–9). Understanding the cumulative progression of the disease over time and the influence of underlying factors is essential. Optimal benefit can only be achieved by identifying and tailoring interventions according to the progressive pathophysiology of CKM syndrome (10).

Modifiable lifestyle factors are key determinants of chronic disease morbidity and mortality and offer the most practical approach to prevention and decelerating disease advancement. Numerous studies have demonstrated that unhealthy lifestyles increase the incidence and mortality of CKD, CVD, and metabolic diseases (11, 12). However, existing studies focus only on a single disease or specific risk factor, with few exploring how multiple risk factors contribute to the dynamic progression of CKM syndrome. Given that CKM syndrome encompasses multiple chronic diseases, favorable lifestyles likely slow its progression and reduce adverse outcomes. Additionally, although many studies have combined lifestyle factors into composite indices to assess their collective association with diseases, only a limited number have systematically quantified the individual contribution of each factor (13).

Therefore, we systematically investigated the relationship between lifestyle and CKM syndrome progression, from early stages (0–3) to clinical CVD stages and death, using multi-state models (MSMs) based on United Kingdom Biobank data. In addition to evaluating the composite lifestyle score, we quantified the relative contributions of individual lifestyle factors to stage advancement. Furthermore, we separately assessed two subtypes of CKM syndrome stage 4, which had not been distinguished in previous studies, and conducted subgroup analyses based on sex and age.

## 2 Materials and methods

### 2.1 Study population

Participants in this study were derived from the United Kingdom Biobank. At baseline, comprehensive data collection included: (1) touchscreen questionnaires assessing socio-demographics, lifestyle factors, and health status; (2) standardized physical examinations; and (3) bio-specimen analyses. These data were subsequently linked to health records for longitudinal outcome monitoring. The United Kingdom Biobank review board granted our study all required approvals (application number 162275). The incident timing of CKM stage-defining events, including CVD, T2D, CKD, renal failure, and death, was precisely ascertained through United Kingdom Biobank follow-ups, enabling the assessment of the transitions of longitudinal disease progression.

**Figure 1:**
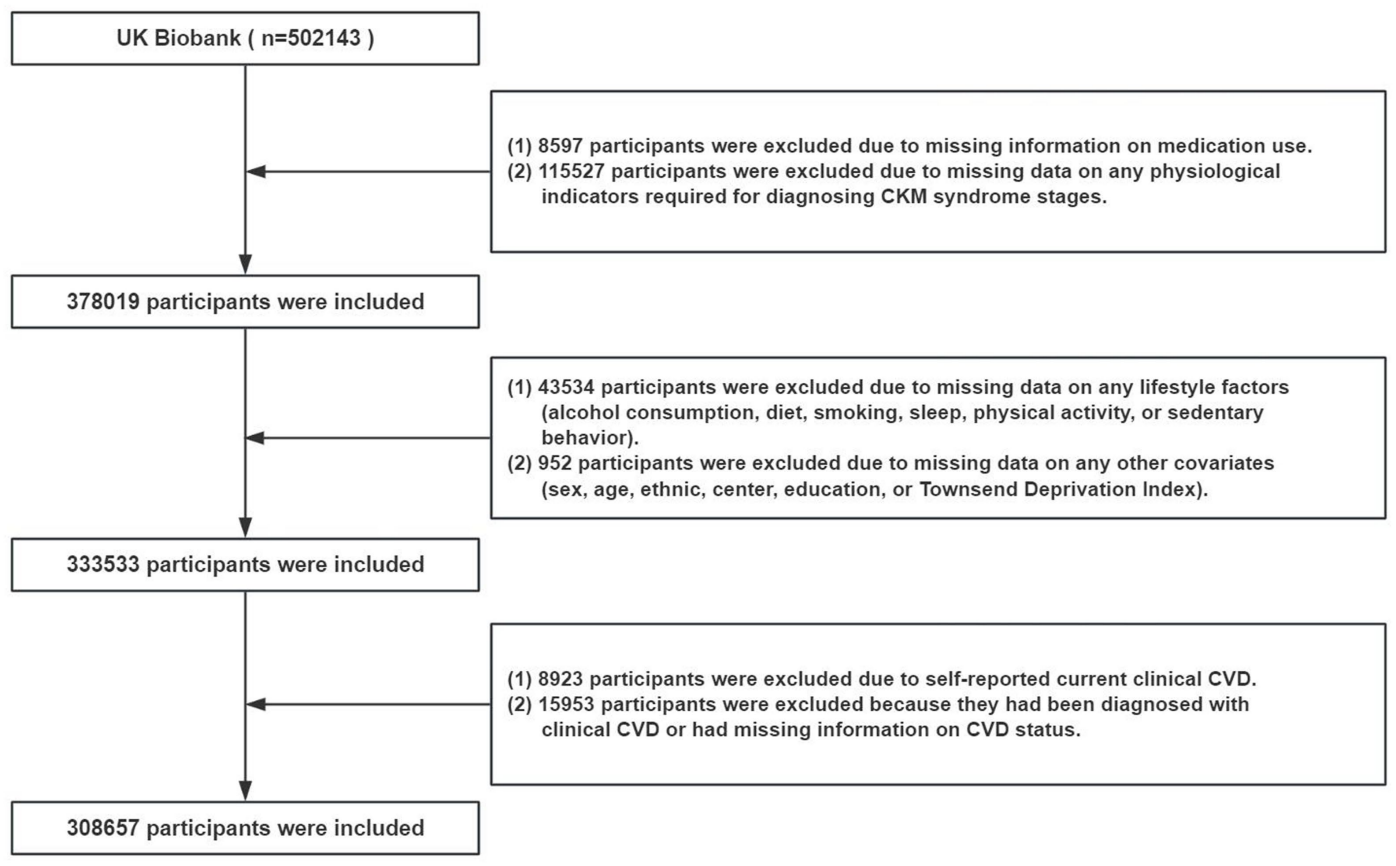
Flowchart for study population selection. CKM, cardiovascular-kidney-metabolic; CVD, cardiovascular disease.

According to the staging criteria for CKM syndrome, we excluded participants with undeterminable disease stages from the initial 502,143 individuals, that is, those missing medication records (*n* = 8,597) or key physiological measurements required (*n* = 115,527). Second, individuals with incomplete lifestyle data (*n* = 43,534) and missing covariate information (*n* = 952) were excluded. Subsequently, because this study focused on individuals with CKM syndrome stages 0–3, those with self-reported (*n* = 8,923) or admission diagnosis of clinical CVD, or missing information on CVD status (*n* = 15,953), were excluded. Ultimately, a total of 308,657 participants were included in the follow-up study (Figure 1).

### 2.2 Definition of CKM syndrome stages 0–3

Based on the AHA Presidential Advisory Statement, CKM syndrome is classified into stages 0 through 4. Stage 0 represents the absence of CKM risk factors, with no evidence of CKD or subclinical/clinical CVD. Stage 1 refers to excessive or dysfunctional obesity with overweight, abdominal obesity, or adipose tissue dysfunction, without accompanying other metabolic risk factors or CKD. Stage 2 encompasses individuals with metabolic risk factors, intermediate to high CKD risk, or both. CKD risk was evaluated based on Kidney Disease: Improving Global Outcomes (KDIGO) (4), with renal function assessed by estimated glomerular filtration rate (eGFR), calculated using the CKD-EPI 2021 equation without the race factor (14). Stage 3 was defined as subclinical CVD, determined by risk equivalents, including extremely high-risk CKD (stage G4 or G5) or a high 10-year CVD risk estimated by the Framingham risk score (15). Stage 4, representing clinical CVD, was considered the subsequent stage of disease progression in this study. As mentioned previously, we excluded those who were at stage 4 at baseline and retained only those at stages 0–3. The specific criteria for determining stages 0–3 of CKM syndrome are provided in Supplementary Table S1.

### 2.3 Determination of disease progression and death outcomes

The early stages (0–3) of CKM syndrome may progress to stage 4 (clinical CVD) and even death, where stage 4 is subdivided into stage 4a (clinical CVD without renal failure) and stage 4b (clinical CVD with renal failure). In the United Kingdom Biobank, we used data collected up to November 30, 2022, with follow-up censored at the earliest occurrence of death, participant withdrawal, or the last available date. Based on prior studies (4, 16), we used the United Kingdom Biobank field codes 41202, 41262, 41270, and 41280. These data were obtained by linking with hospital admission records, with new-onset clinical CVD and renal failure diagnoses based on the corresponding International Classification of Diseases, 10th Revision codes. Death data were obtained from death certificates provided by the National Health Service (NHS) Information Center (England and Wales) and the NHS Central Register (Scotland). Detailed determinations of disease progression are shown in Supplementary Table S2.

### 2.4 Assessment of lifestyle factors

Informed by prior research, we considered four traditional factors (smoking, alcohol consumption, physical activity, and diet) along with two emerging factors (sleep duration and sedentary behavior) (17, 18). All lifestyle factors were collected through validated touchscreen questionnaires, and details of the assessment are provided in Supplementary Table S3.

In the United Kingdom Biobank, smoking status was classified into three distinct categories: current, previous, and never, with the latter group regarded as low-risk (16). Alcohol consumption was self-reported, based on weekly or monthly intake of alcoholic beverages. Reported intake was standardized according to the latest NHS recommendations, with one standard serving defined as 8 g (10 mL) of pure alcohol. Low-risk alcohol consumption was identified as relatively regular moderate drinking (up to 14 units per week) (19). A low-risk diet met at least four of seven recommended dietary criteria: increased intake of fruits, vegetables, whole grains, and fish, along with reduced consumption of refined grains, processed meats, and unprocessed red meats. The conversion method for food group serving sizes was based on prior United Kingdom Biobank studies (20). Regular physical activity was defined as engaging in ≥150 min of moderate activity per week, or ≥75 min of vigorous activity per week, or an equal amount of a combination of moderate and vigorous activity. Sleep duration was assessed by a sleep questionnaire, defining 7–9 h as low-risk sleep (17). Sedentary behavior was measured by self-reported total sedentary time, including time spent on watching television and using computers for leisure (excluding work use), with low-risk defined as <4 h/day (16). For each factor, a score of 1 was assigned to the low-risk and 0 to the higher-risk. The lifestyle score was calculated by summing these values and subsequently divided into three groups for analysis: Low (0-1), Medium (2–4), and High (5-6), with higher scores reflecting better adherence to a healthy lifestyle.

### 2.5 Ascertainment of covariates

In line with previous studies (17, 18), we adjusted for potential confounders, including demographic characteristics and socioeconomic indicators: age, gender, ethnic background, education, Townsend deprivation index (TDI), and assessment center. Baseline metabolic and clinical indicators were not included in the primary models because these variables may partly reflect intermediate processes linking lifestyle behaviors to CKM progression. Adjusting for such factors could therefore lead to over-adjustment and attenuation of the associations of interest. To evaluate the robustness of the findings and address potential confounding by baseline disease severity, staged-adjustment sensitivity analyses were conducted by further adjusting for baseline CKM stage severity or selected metabolic risk indicators.

### 2.6 Statistical analyses

First, baseline characteristics were described for all participants and across three lifestyle groups. Categorical variables were presented as *n* (%), with group differences assessed using the *χ*^2^ test. According to the Kolmogorov–Smirnov test (Supplementary Table S4), all continuous variables deviated from normality and were therefore presented as medians with interquartile ranges (IQRs), and the Kruskal–Wallis test was applied for group comparisons.

We then fitted traditional Cox proportional hazards models to estimate the associations between the composite lifestyle score and individual lifestyle factors with the status of CKM syndrome progression (including overall stage 4, stage 4a, stage 4b, and all-cause mortality). Two levels of adjustment were consistently applied in both the basic Cox analyses and subsequent MSMs: Model 1 adjusted for age and sex, while Model 2 further included ethnic background, TDI, education, and assessment center. When analyzing individual lifestyle factors, all six were mutually adjusted in the models. The Schoenfeld residuals plots were used to evaluate the proportional hazards assumption, and no substantial violations were observed.

According to the advancement patterns, we used MSMs to examine the independent and joint contributions of lifestyle factors to CKM syndrome progression from stages 0–3 to clinical stages and death. Multi-state models synthesize concepts from competing risks analysis and Cox regression, enabling the quantification of factor effects on each possible transition pathway in staged disease progression systems like CKM syndrome. (21). In our initial analyses, we developed an MSM for the longitudinal progression from stages 0–3 to CKM stage 4 and all-cause death, termed transition pattern A. Three transitions were included: (1) baseline to CKM stage 4, (2) baseline to death, and (3) CKM stage 4 to death. Results were presented as hazard ratios (HRs) and 95% confidence intervals (CIs).

All multi-state models were fitted using the “mstate” package. Follow-up time from study entry was used as the timescale, with censoring handled as described in the Study Population section. To allow covariate effects to differ across transitions, lifestyle score and other covariates were expanded using the “expand.covs” function. The expanded dataset was then fitted using the “coxph” function, stratifying by transition. The proportional hazards assumption was tested for each transition using Schoenfeld residuals, while no significant violations were detected. Further details of the MSM specification are provided in Supplementary Method S1.

The lifestyle score was calculated by equally weighting each component, a method widely adopted in epidemiologic studies despite the inherent assumption that all factors exert the same impact on outcomes. To address this limitation, we further applied the quantile G-computation (QGC) to quantify the relative contributions of individual lifestyle factors at each transition in CKM progression (see Supplementary Method S2). The QGC method, widely applied in epidemiologic research (16), was employed to infer causality and to estimate the positive or negative relative contributions of lifestyle components. In addition, we calculated the population-attributable risk (PAR) to estimate the theoretically preventable proportion of each CKM syndrome transition under optimal adherence to a healthy lifestyle (score 5–6) (16). Assuming that lifestyle factors causally influence disease progression, we conducted 500 bootstrap resampling, calculating PAR at each iteration. The 2.5th and 97.5th percentile values were used to construct 95% CI, while the mean PAR was taken as the point estimate (22). Given the relative stability of PAR estimates across time horizons from 1 to 10 years, the 5-year risk was selected as the primary outcome for analysis.

Building on the initial findings, we refined the classification of CKM syndrome stage 4 and constructed a new MSM for primary analyses. This updated model offers a more comprehensive and granular depiction of CKM progression, encompassing six transitions within transition pattern B: (1) baseline → CKM stage 4a, (2) baseline → CKM stage 4b, (3) baseline → death, (4) CKM stage 4a → CKM stage 4b, (5) CKM stage 4a → death, and (6) CKM stage 4b → death. The QGC method was again applied to estimate the relative contributions, and PARs were similarly calculated. In all MSMs, for participants (*n* = 37) who transitioned into multiple states on the same day, the entry date for the earlier state was set to 0.5 days prior to that of the subsequent state (23).

We further explored potential effect modification by baseline age (<60 vs. ≥60 years) and sex (male vs. female) through stratified analyses and interaction tests (24). Specifically, MSMs were fitted within each stratum, and the main analyses were repeated. Interaction effects were evaluated using likelihood ratio tests by comparing models with and without cross-product terms.

The robustness of the findings was assessed through several sensitivity analyses following the refinement of the CKM stage 4 classification. First, to mitigate potential reverse causality, outcome events that occurred within the initial two-year follow-up period were excluded. Second, to assess the impact of interval assumptions, the entry date for participants transitioning into different stages on the same date was recalculated using alternative time intervals (1, 3, and 5 days). Third, individuals who entered different stages on the same date were excluded. In addition, to evaluate whether the observed associations were influenced by baseline disease severity or metabolic status, staged-adjustment sensitivity analyses were conducted. Specifically, the main models were further adjusted for baseline CKM stage severity. In a separate model, additional adjustment was made for key metabolic indicators, including body mass index (BMI), systolic blood pressure, LDL cholesterol, and fasting glucose. R software (v4.4.2) was employed for all statistical analyses, considering a two-sided *p*-value <0.05 as statistically significant.

## 3 Results

### 3.1 Participant characteristics

Among 308,657 participants classified as stages 0–3 of CKM syndrome, 27,132 were categorized into the Low lifestyle score group, 220,118 into the Medium group, and 61,407 into the High group. Significant differences in age [median (IQR), 57 (50–63) years; *p* < 0.001] and gender [138,359 males (44.83%) and 170,298 females (55.17%); *p* < 0.001] were observed across lifestyle subgroups. The majority of participants (95.72%) were White. As the composite lifestyle score increased, the TDI decreased, and educational attainment increased. In other words, individuals with lower lifestyle scores tended to be older, less educated males with higher TDI values. Furthermore, higher adherence to a variety of healthy lifestyle behaviors correlated with earlier CKM syndrome stages, with only 15.02% of participants in the High group being in stage 3, compared to 53.87% in the Low group. These findings indicate that lower lifestyle scores were associated with more advanced CKM stages at baseline. (Table 1).

**Table 1:** Baseline characteristics by lifestyle score level among individuals in CKM stages 0–3.

Characteristics	Total	Lifestyle score			*p*
		Low [0-1]	Medium [2–4]	High [5-6]	
*N*	308,657	27,132	220,118	61,407	
Age, year	57 (50–63)	58 (51–63)	57 (50–63)	56 (49–62)	<0.001
Gender (%)					<0.001
Male	138,359 (44.83)	16,224 (59.80)	102,735 (46.67)	19,400 (31.59)	
Female	170,298 (55.17)	10,908 (40.20)	117,383 (53.33)	42,007 (68.41)	
Ethnic background (%)					<0.001
White	295,446 (95.72)	25,977 (95.74)	210,184 (95.49)	59,285 (96.54)	
Non-White	13,211 (4.28)	1,155 (4.26)	9,934 (4.51)	2,122 (3.46)	
Education (%)					<0.001
No qualification	42,489 (13.77)	6,237 (22.99)	31,377 (14.25)	4,875 (7.94)	
Any other qualifications	158,500 (51.36)	14,741 (54.33)	114,824 (52.16)	28,935 (47.12)	
College or university degree	107,668 (34.87)	6,154 (22.68)	73,917 (33.58)	27,597 (44.94)	
TDI	−2.30 (−3.71–0.15)	−1.53 (−3.32–1.64)	−2.27 (−3.7–0.19)	−2.61 (−3.89−−0.58)	<0.001
Lifestyle factors (%)					
Low-risk alcohol consumption	172,096 (55.75)	2042 (7.53)	83,895 (38.11)	49,624 (80.81)	<0.001
Low-risk diet	162,115 (52.53)	2025 (7.46)	92,838 (42.18)	51,679 (84.16)	<0.001
Never smoking	136,265 (44.15)	3,213 (11.84)	114,925 (52.21)	54,254 (88.35)	<0.001
Low-risk sleep	77,899 (25.23)	9,207 (33.93)	163,799 (74.41)	57,752 (94.05)	<0.001
Regular physical activity	127,940 (41.45)	4,094 (15.09)	122,531 (55.67)	54,092 (88.09)	<0.001
Low-to-moderate sedentary behavior	146,590 (47.49)	2,311 (8.52)	106,140 (48.22)	53,616 (87.31)	<0.001
CKM stages (%)					<0.001
0	27,360 (8.87)	914 (3.37)	17,334 (7.87)	9,112 (14.84)	
1	21,563 (6.99)	1,216 (4.48)	15,061 (6.84)	5,286 (8.61)	
2	163,266 (52.89)	10,387 (38.28)	115,095 (52.29)	37,784 (61.53)	
3	96,468 (31.25)	14,615 (53.87)	72,628 (33.00)	9,225 (15.02)	

CKM, cardiovascular-kidney-metabolic; TDI, Townsend deprivation index. Continuous variables are summarized using median (quantile1, quantile3), and categorical variables as number (%).

**Figure 2:**
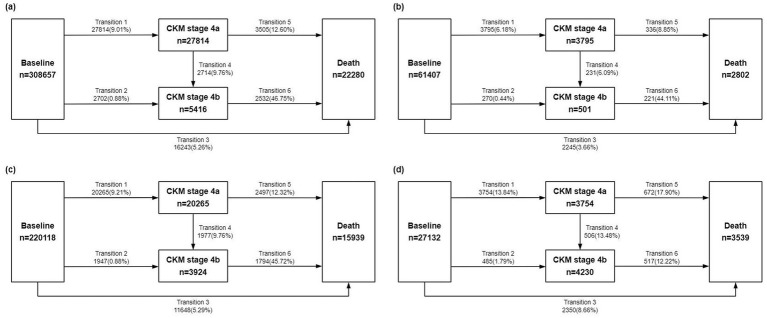
Numbers (percentages) of participants in CKM transition pattern B by lifestyle score: **(a)** Overall population; **(b)** Lifestyle score 5–6 group; **(c)** Lifestyle score 2–4 group; **(d)** Lifestyle score 0–1 group. CKM, cardiovascular-kidney-metabolic.

With a median follow-up duration of 13.71 years (IQR: 13.01–14.38 years), a total of 30,516 participants progressed to CKM stage 4, and 22,280 deaths occurred. Supplementary Figure S1 demonstrates the number and proportions of participants progressing through transition pattern A. It shows that 9.89% of participants progressed to stage 4, while 19.87% died after progression and 5.26% died without reaching stage 4. Details of transition pattern B are presented in Figure 2. A total of 27,814 participants (9.01%) experienced stage 4a, of which 2,714 (9.76%) subsequently progressed to stage 4b, while an additional 2,702 skipped stage 4a and advanced directly. Overall, the proportion of direct deaths was 5.26% among all participants, 12.60% among those in stage 4a, and 46.75% among those in stage 4b. Higher lifestyle scores were associated with lower rates of progression to stage 4 (including both 4a and 4b) and death. Thus, Participants who progressed to stage 4 or died tended to have lower lifestyle scores and a higher number of unhealthy lifestyle behaviors.

### 3.2 Association between lifestyle and CKM syndrome progression: initial findings

First, we explored the associations between the composite lifestyle score, individual lifestyle factors, and each disease state using Cox regression models. Healthier lifestyle metrics consistently correlated with lower risks of stage 4 (total/4a/4b) and death (Table 2).

**Table 2:** Associations of lifestyle with the risks of total CKM stage 4, CKM stage 4a, CKM stage 4b, and death using the Cox model.

Characteristics	CKM stage 4		CKM stage 4a		CKM stage 4b		Death	
	Model 1	Model 2	Model 1	Model 2	Model 1	Model 2	Model 1	Model 2
Lifestyle score								
[0–1]	1	1	1	1	1	1	1	1
[2–4]	0.68 (0.65, 0.70)	0.72 (0.70, 0.74)	0.70 (0.68, 0.72)	0.74 (0.72, 0.77)	0.53 (0.49, 0.56)	0.59 (0.55, 0.63)	0.58 (0.56, 0.61)	0.63 (0.61, 0.65)
[5–6]	0.49 (0.47, 0.52)	0.56 (0.53, 0.58)	0.52 (0.50, 0.55)	0.58 (0.56, 0.61)	0.28 (0.25, 0.31)	0.34 (0.31, 0.38)	0.41 (0.39, 0.43)	0.46 (0.44, 0.49)
Per score point	0.85 (0.84, 0.85)	0.87 (0.86, 0.87)	0.86 (0.85, 0.87)	0.88 (0.87, 0.89)	0.74 (0.73, 0.76)	0.78 (0.76, 0.79)	0.81 (0.80, 0.82)	0.83 (0.83, 0.84)
Lifestyle factors								
Low-risk alcohol consumption	0.87 (0.85, 0.89)	0.90 (0.88, 0.92)	0.88 (0.86, 0.90)	0.90 (0.88, 0.92)	0.79 (0.75, 0.84)	0.82 (0.78, 0.87)	0.84 (0.82, 0.87)	0.86 (0.84, 0.89)
Low-risk diet	0.89 (0.87, 0.91)	0.92 (0.90, 0.94)	0.90 (0.88, 0.93)	0.93 (0.91, 0.95)	0.77 (0.73, 0.82)	0.81 (0.77, 0.86)	0.84 (0.82, 0.87)	0.87 (0.85, 0.90)
Never smoking	0.76 (0.74, 0.77)	0.78 (0.76, 0.79)	0.77 (0.75, 0.79)	0.79 (0.77, 0.81)	0.64 (0.60, 0.68)	0.68 (0.64, 0.72)	0.66 (0.64, 0.68)	0.69 (0.67, 0.71)
Low-risk sleep	0.82 (0.80, 0.85)	0.85 (0.83, 0.87)	0.83 (0.81, 0.86)	0.86 (0.84, 0.88)	0.75 (0.71, 0.79)	0.80 (0.76, 0.85)	0.85 (0.82, 0.87)	0.88 (0.85, 0.90)
Regular physical activity	0.89 (0.87, 0.91)	0.89 (0.87, 0.91)	0.91 (0.89, 0.93)	0.91 (0.89, 0.93)	0.74 (0.70, 0.78)	0.74 (0.70, 0.78)	0.83 (0.81, 0.86)	0.84 (0.82, 0.86)
Low-to-moderate sedentary behavior	0.85 (0.83, 0.87)	0.87 (0.85, 0.89)	0.86 (0.84, 0.88)	0.88 (0.86, 0.91)	0.79 (0.74, 0.83)	0.82 (0.78, 0.87)	0.87 (0.84, 0.89)	0.90 (0.87, 0.92)

CKM stage 4 comprises CKM stage 4a and CKM stage 4b. Model 1 was adjusted for age and sex; model 2 was further adjusted by adding ethnic background, TDI, education, and assessment center. For analyses on individual lifestyle factors, all lifestyle factors were simultaneously adjusted. All *p*-values were <0.001.

The MSM analyses of lifestyle score, lifestyle factors, and transition pattern A (without subtype differentiation) are shown in Supplementary Table S5 (Model 1) and Table 3 (Model 2). Among the three transitions, compared with participants scoring 0-1, participants adhering to 2–4 healthy lifestyle behaviors had reduced risks by 28% [HR = 0.72 (95% CI: 0.70–0.74)], 35% [HR = 0.65 (95% CI: 0.62–0.68)], and 30% [HR = 0.70 (95% CI: 0.66–0.75)], respectively. Those adhering to 5-6 healthy lifestyle behaviors showed even greater reductions of 44% [HR = 0.56 (95% CI: 0.53–0.58)], 50% [HR = 0.50 (95% CI: 0.47–0.53)] and 48% [HR = 0.52 (95% CI: 0.47–0.57)], respectively. For each 1-point increase in lifestyle score, the fully adjusted HRs were 0.87 (95% CI: 0.86–0.87), 0.85 (95% CI: 0.84–0.86), and 0.86 (95% CI: 0.84–0.88), respectively. Participants with the highest adherence to a healthy lifestyle (5-6) had PARs of 29.97, 31.53, and 39.97% for each transition compared to those with relatively poor adherence (0–4), representing the proportion of events that might theoretically be attributable to lifestyle differences under model assumptions. Additionally, every healthy lifestyle factor was associated with a decreased risk of all CKM syndrome transitions.

**Table 3:** Associations between lifestyle and risks of transitions in pattern A using multi-state model (model 2).

Characteristics	Baseline → CKM stage 4		Baseline → death		CKM stage 4 → death	
	HR (95% CI)	*p*	HR (95% CI)	*p*	HR (95% CI)	*p*
Lifestyle score						
[0–1]	1	—	1	—	1	—
[2–4]	0.72 (0.70, 0.74)	<0.001	0.65 (0.62, 0.68)	<0.001	0.70 (0.66, 0.75)	<0.001
[5-6]	0.56 (0.53, 0.58)	<0.001	0.50 (0.47, 0.53)	<0.001	0.52 (0.47, 0.57)	<0.001
Per score point	0.87 (0.86, 0.87)	<0.001	0.85 (0.84, 0.86)	<0.001	0.86 (0.84, 0.88)	<0.001
PAR (%)	29.97 (30.30, 29.66)	—	31.53 (31.86, 31.21)	—	39.97 (40.70, 39.20)	—
Lifestyle factors						
Low-risk alcohol consumption	0.90 (0.88, 0.92)	<0.001	0.87 (0.84, 0.90)	<0.001	0.89 (0.84, 0.94)	<0.001
Low-risk diet	0.92 (0.90, 0.94)	<0.001	0.89 (0.86, 0.92)	<0.001	0.88 (0.83, 0.92)	<0.001
Never smoking	0.78 (0.76, 0.79)	<0.001	0.73 (0.70, 0.75)	<0.001	0.68 (0.65, 0.72)	<0.001
Low-risk sleep	0.85 (0.83, 0.87)	<0.001	0.89 (0.86, 0.92)	<0.001	0.96 (0.91, 1.02)	0.213
Regular physical activity	0.89 (0.87, 0.91)	<0.001	0.86 (0.83, 0.88)	<0.001	0.86 (0.82, 0.90)	<0.001
Low-to-moderate sedentary behavior	0.87 (0.85, 0.89)	<0.001	0.91 (0.88, 0.94)	<0.001	0.93 (0.88, 0.98)	0.007

HR, hazard ratio; CI, confidence interval; —, not applicable (reference group or statistic not calculated). The model was adjusted for age, sex, ethnic background, TDI, education, and assessment center.

Supplementary Figure S2 (Model 1) and Supplementary Figure S3 (Model 2) show the QGC results for each lifestyle factor. Never smoking made the largest contribution to delaying disease progression, with contributions of 29.5%, 33.6%, and 42.6%, respectively.

### 3.3 Results after subclassification of CKM stage 4 into 4a and 4b

Based on transition pattern A, we further subdivided stage 4 into two subtypes: stage 4a and stage 4b (transition pattern B). The associations between the lifestyle score and the six transitions are presented in Supplementary Table S6 (Model 1) and Table 4 (Model 2). In general, the lifestyle score was associated with all transitions, but to varying degrees. Adhering to a healthy lifestyle showed the strongest association with lower risk of transition from baseline to stage 4b, with a HR of 0.57 (95% CI: 0.52–0.63) in the score 2–4 group and 0.35 (95% CI: 0.30–0.41) in the score 5-6 group, in comparison to the 0-1 score group. After full adjustment, the HR per 1-point increase in lifestyle score was 0.77 (95% CI: 0.75–0.79). For nearly all transitions, the correlation was stronger for higher score groups, with greater risk reduction. Although no statistically significant results were observed for participants scoring 5-6 during the transition from stage 4b to death, this may be attributed to the limited number of cases. Furthermore, participants with the highest adherence to a healthy lifestyle (5-6) had the largest PAR (67.76%) for the transition from baseline to CKM stage 4b, and the smallest PAR (2.64%) for the transition from stage 4b to death.

**Table 4:** Associations between lifestyle score and risks of transitions in pattern B using multi-state model (model 2).

Transitions	Low [0-1]		Medium [2–4]		High [5-6]		Per score point		PAR (%)
	HR (95% CI)	*p*	HR (95% CI)	*p*	HR (95% CI)	*p*	HR (95% CI)	*p*	
Baseline → CKM stage 4a	1	—	0.74 (0.71, 0.77)	<0.001	0.58 (0.56, 0.61)	<0.001	0.88 (0.87, 0.89)	<0.001	27.32 (27.03, 27.64)
Baseline → CKM stage 4b	1	—	0.57 (0.52, 0.63)	<0.001	0.35 (0.30, 0.41)	<0.001	0.77 (0.75, 0.79)	<0.001	67.76 (67.09, 68.35)
Baseline → death	1	—	0.65 (0.62, 0.68)	<0.001	0.50 (0.47, 0.53)	<0.001	0.85 (0.84, 0.86)	<0.001	31.53 (31.21, 31.86)
CKM stage 4a → CKM stage 4b	1	—	0.75 (0.68, 0.82)	<0.001	0.49 (0.42, 0.57)	<0.001	0.86 (0.84, 0.89)	<0.001	54.02 (53.15, 54.91)
CKM stage 4a → death	1	—	0.71 (0.65, 0.77)	<0.001	0.52 (0.46, 0.60)	<0.001	0.86 (0.84, 0.89)	<0.001	38.97 (38.16, 39.83)
CKM stage 4b → death	1	—	0.85 (0.77, 0.94)	0.002	0.86 (0.73, 1.01)	0.066	0.96 (0.93, 0.99)	0.008	2.64 (1.97, 3.30)

HR, hazard ratio; CI, confidence interval; —, not applicable (reference group). The model was adjusted for age, sex, ethnic background, TDI, education, and assessment center.

**Figure 3:**
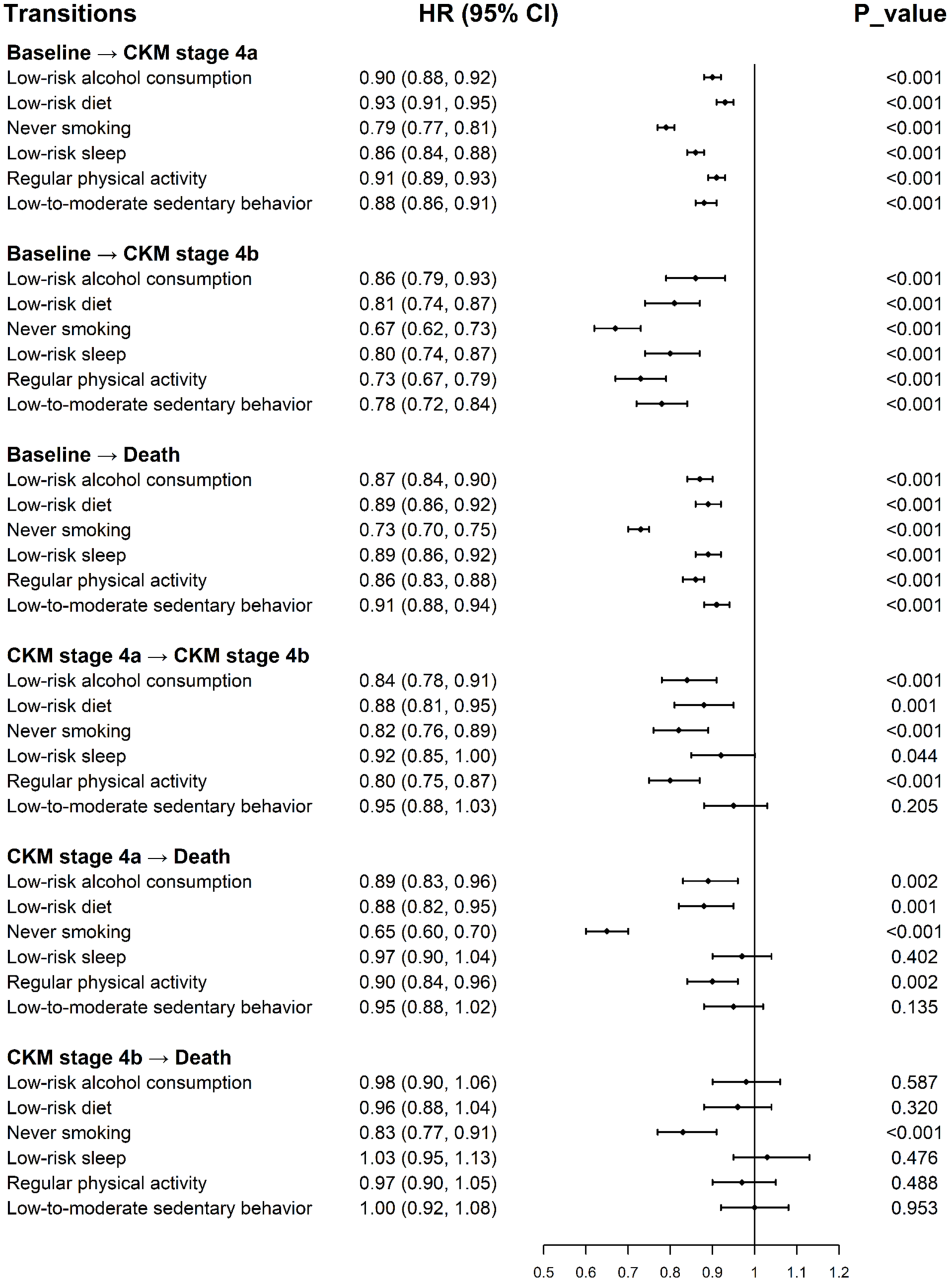
The role of lifestyle factors in transition pattern B of CKM syndrome (model 2). HR, hazard ratio; CI, confidence interval; CKM, cardiovascular-kidney-metabolic. Model was adjusted for age, sex, ethnic background, TDI, education, and assessment center.

Supplementary Figure S4 (Model 1) and Figure 3 (Model 2) illustrate the associations of lifestyle factors with every transition. Each low-risk lifestyle factor was significantly associated with a slower progression of CKM syndrome. For instance, for transitions from baseline to CKM stage 4a, CKM stage 4b, and death, the fully adjusted HRs for never smoking were 0.79 (95% CI: 0.77–0.81), 0.67 (95% CI: 0.62–0.73), and 0.73 (95% CI: 0.70–0.75), respectively. As CKM syndrome advanced to later stages, the magnitude of the associations between lifestyle factors and transition risks became smaller. Specifically, during the transition from baseline to any subsequent stage, all six healthy lifestyle factors demonstrated reduced transition risks. In the transition from stage 4a to stage 4b, five factors—excluding low-to-moderate sedentary behavior—continued to confer a protective effect. In the transition from stage 4a to death, only four lifestyle factors, namely low-risk alcohol consumption, low-risk diet, never smoking, and regular physical activity, remained significantly associated with a lower risk of progression. However, even in the most terminal stage 4b to death transition, an HR of 0.83 (95% CI: 0.77–0.91) was still observed for never smoking.

**Figure 4:**
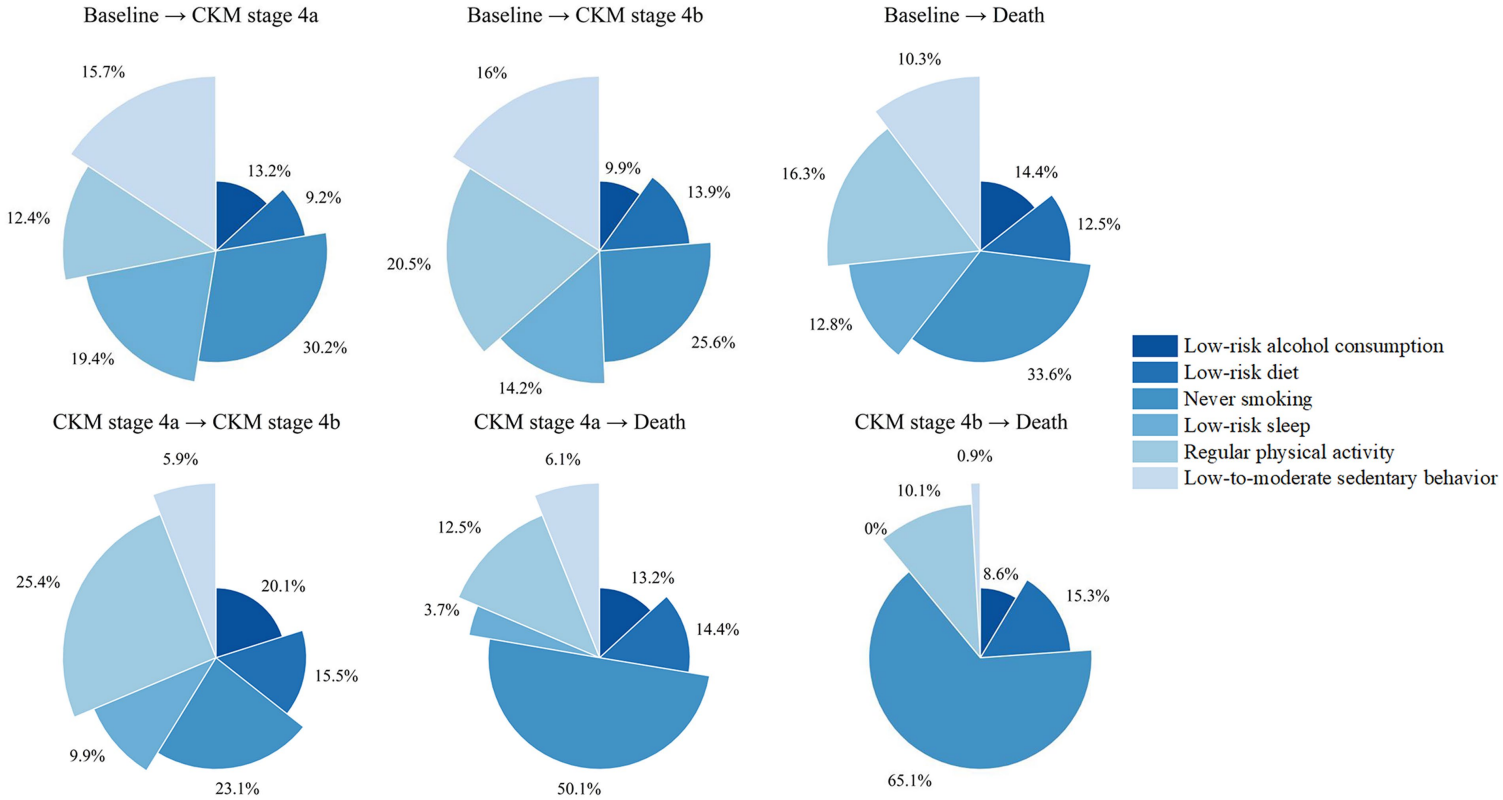
Relative contributions of lifestyle factors to transition pattern B (model 2). Quantile G-computation was used to quantify the relative contributions of lifestyle factors to CKM progression. The segments illustrate the contributions of each factor. Model was adjusted for age, sex, ethnic background, TDI, education, and assessment center.

The relative contributions of each lifestyle factor to the six transitions are shown in Supplementary Figure S5 (Model 1) and Figure 4 (Model 2). Among all factors, never smoking showed the largest relative contribution to the observed transition risk differences, with contribution values ranging from 23.1 to 65.1%. There is some overlap between the top three contributors in each conversion. For example, apart from never smoking, for the transition from baseline to stage 4a, low-risk sleep (19.4%) and low-to-moderate sedentary behavior (15.7%) were also major contributors, while for the transition from baseline to CKM stage 4b, regular exercise (20.5%) and low-to-moderate sedentary behavior (16.0%) were among the top contributors.

### 3.4 Results of subgroup and sensitivity analyses

Subgroup analyses, based on baseline age and sex, were conducted to explore the association between lifestyle score and CKM syndrome transition pattern B. The results were generally consistent across all subgroups. Although several statistically significant interactions were observed, most were considered clinically meaningless (Supplementary Tables S7–S10). Lifestyle score was more strongly associated with younger participants and female participants than their older and male counterparts. The robustness of our results was confirmed through serial sensitivity analyses, which included the exclusion of outcome events within the first 2 years (Supplementary Table S11), the assignment of distinct time intervals when participants entered different stages on the same date (Supplementary Tables S12–S14), and the exclusion of participants with simultaneous state entries (Supplementary Table S15). In addition, staged-adjustment sensitivity analyses were conducted to account for baseline disease severity and metabolic status. After additional adjustment for baseline CKM stage (Supplementary Table S16) or for major metabolic indicators including BMI, systolic blood pressure, LDL cholesterol, and fasting glucose (Supplementary Table S17), the associations between lifestyle score and CKM progression remained largely consistent in direction and magnitude, with only modest attenuation of effect estimates.

## 4 Discussion

In this prospective study, we systematically examined, for the first time, the association of overall lifestyle score and individual lifestyle factors with longitudinal transitions in CKM syndrome, and further explored the relative contributions of individual factors, including alcohol consumption, diet, smoking, physical activity, sleep duration, and sedentary behavior. All six lifestyle factors, along with their composite score, were consistently associated with lower risks of CKM syndrome transitions, including transitions related to stage 4a, stage 4b, total stage 4, and subsequent death. The protective effects strengthened with higher lifestyle scores, with approximately a 15% reduction in the risk of disease progression for each additional behavior followed. Notably, the protective effects progressively weakened as CKM syndrome advanced, especially after entering stage 4b, where mortality rates increased substantially. This trend highlights the critical need for early intervention. Of all transitions, the transition from baseline to stage 4b was most strongly associated with healthy lifestyle adherence, with a 65% lower risk observed among participants adhering to 5-6 healthy behaviors, compared to those adhering to 0-1. Among the lifestyle factors, never smoking emerged as the most influential protective factor overall. Even when full adherence was unattainable, adhering to four core behaviors (moderate alcohol consumption, high-quality diet, never smoking, and regular physical activity) was still associated with substantially lower transition risks.

Previous epidemiologic studies have extensively demonstrated significant associations between a healthy lifestyle and the risk of events such as CVD, type 2 diabetes, and CKD (18, 25). Nevertheless, most existing research has focused exclusively on individual diseases rather than considering their co-development. As pivotal elements of CKM syndrome, these diseases elevate the risk of death independently and further exacerbate the burden when they occur together. For instance, patients with type 2 diabetes and coexisting CKD and CVD had nearly a fivefold higher risk of all-cause mortality than those without complications, according to a large cohort study (26). Although some studies have examined the effect of lifestyle on cardiac, renal, and metabolic multi-morbidity and disease accumulation sequences, they primarily focus on the number of comorbidities rather than the disease advancement within an integrated pathophysiological framework (16, 27). Moreover, current evidence on CKM syndrome predominantly derives from cross-sectional studies examining mortality and prevalence rates, with limited attention to the longitudinal evolution. For example, a study covering 3,101 counties in the US reported that the pooled median county-level age-adjusted mortality rate was 505.5 per 100,000 residents (IQR: 441.3–578.9) between 2010 and 2019 (28). Unlike traditional Cox models, MSMs offer a superior approach by dynamically capturing phase transitions, which is particularly critical for progressive conditions like CKM syndrome. Therefore, this study provides the first dynamic longitudinal analysis of CKM syndrome within a holistic multi-system framework, systematically delineating its progression pathway by MSMs and emphasizing the crucial role of lifestyle factors in mitigating disease advancement.

In line with prior evidence, our study confirmed elevated mortality in CKM stage 4, underscoring the synergistic interplay of metabolic, cardiovascular, and renal impairments within the CKM syndrome framework (3, 7). After a follow-up of at least 13 years, approximately 10% of our study population progressed to CKM stage 4, with CKM stage 4a being the most prevalent. However, few studies have considered the clinical heterogeneity of CKM stage 4. Stage 4 represents a critical phase of clinical CVD, coexisting with varying degrees of renal dysfunction, and significant differences in pathophysiologic mechanisms, disease trajectories, and therapeutic strategies may exist (4). Thus, a finer classification holds substantial clinical relevance. Previous studies have suggested that CKM syndrome and kidney injury are mutually reinforcing (29, 30), and our findings corroborate this, showing that stage 4b (with renal failure) carries a significantly higher mortality risk than stage 4a (without renal failure). Notably, stage 4b was identified as the worst stage for disease progression. This may be due to the complex cardiorenal interactions of endocrine abnormalities, urotoxin accumulation, and hemodynamic alterations triggered by renal failure (31). Therefore, delaying CKM syndrome progression from a state without renal failure to stage 4b warrants prioritized investigation. We observed that higher lifestyle scores were strongly associated with a lower risk of transition to stage 4b, with the greatest individual and collective benefits (PAR: 67.76%) achieved when timely interventions were implemented.

Our study also supports previous findings that never smoking is the most significant protective factor (16, 32). This lifestyle factor impacts all transitions in CKM syndrome, suggesting that smoking status may be an important factor associated with CKM progression. Mechanistically, smoking contributes to oxidative stress, endothelial dysfunction, inflammation, and lipid modifications that promote atherosclerosis and lead to CVD (33). Moreover, smoking exacerbates genetic susceptibility to diabetes and leads to damage in pancreatic tissue and dysfunction of β-cells directly (34). The presence of glycotoxins in cigarettes promotes the formation of advanced glycation end products, which compromise vascular permeability and induce pathological vascular changes, thereby increasing the risk of CKD among smokers (both current and former) compared to never-smokers (35). Physical activity contributed more than never smoking to the transition from CKM stage 4a to 4b. This finding suggests that among individuals with established CVD, regular physical activity may play a key role in preventing progression to end-stage cardiovascular-renal comorbidity. Compared with never smoking, which represents a preventive behavior, regular physical activity may act as a more proactive strategy that promotes coordinated improvements in both cardiovascular and renal systems (36, 37). Consistent with this, a recent study reported that exercise training provides physiological benefits across the full CKM spectrum (38), and our findings further highlight its contribution to transitions within advanced CKM stages. In addition, other lifestyle factors investigated in the present study likewise had protective effects against the CKM syndrome, consistent with prior epidemiological evidence (9, 16, 39). Portfolio analyses revealed synergistic effects among the six factors, with combined lifestyle scores significantly enhancing protection against disease progression and mortality.

This study represents a novel attempt to quantitatively disentangle individual risk factor contributions in CKM syndrome, establishing a framework for prioritizing interventions. Beyond conventional lifestyle factors, our study incorporated emerging determinants (sleep duration and sedentary behavior) and further provided complementary evidence supporting their protective roles against CKM syndrome progression (18, 40). Moreover, we found that adherence to just four key behaviors (moderate alcohol consumption, high-quality diet, never smoking, and regular exercise) still provided significant protection, given their particularly robust and consistent protective effects throughout disease transitions. This finding aligns with global prevention strategies. As outlined in the World Health Organization’s Global Action Plan for the Prevention and Control of Non-Communicable Diseases, tobacco use, unhealthy diet, harmful use of alcohol, and lack of physical activity are key risk factors requiring urgent intervention (41). Given that existing CKM studies have primarily focused on populations in the United States and China, our study’s focus on a United Kingdom cohort provides a valuable complement to understanding CKM progression and intervention, contributing to the global consensus on CKM management.

There are several limitations to acknowledge. First, the absence of cardiac biomarkers, echocardiographic data, and coronary angiography in the United Kingdom Biobank for defining stage 3 CKM may have led to underestimation of disease staging, despite the application of risk equivalents derived from AHA guidelines and previous studies (4, 42). Second, selection bias may be present because a substantial number of participants were excluded during the cohort selection process. In large cohort studies such as the United Kingdom Biobank, missingness may not be completely random and may be associated with socioeconomic status, health conditions, or behavioral characteristics. To evaluate this possibility, we compared the baseline characteristics of included and excluded participants (Supplementary Table S16). Although some differences were observed, the overall patterns were generally comparable. Nevertheless, the potential influence of selection bias on the study findings cannot be entirely excluded. Third, lifestyle factors were assessed through self-reported questionnaires, which may introduce recall bias and exposure misclassification. In large prospective cohort studies such as the United Kingdom Biobank, self-reported lifestyle information is commonly used and has been shown to provide reasonably valid estimates for population-level associations, although some degree of measurement error is unavoidable. Fourth, lifestyle behaviors were measured only at baseline, and therefore, changes in behaviors during follow-up could not be captured. Such time-varying changes may lead to non-differential exposure misclassification, which would generally bias the estimated associations toward the null rather than inflate the observed effects. Nevertheless, restricting exposure assessment to baseline may also reduce potential reverse causality arising from lifestyle modifications after disease diagnosis. Fifth, for participants undergoing same-date stage transitions, we applied a 0.5-day interval adjustment to assign entry dates, which may introduce inaccuracies. However, sensitivity analyses with varying time intervals confirmed the robustness of effect estimates. Sixth, residual confounding by unexplained factors, such as genetic susceptibility and environmental exposures, may remain despite reasonable adjustments for multiple covariates. In particular, baseline CKM stage differed across lifestyle groups, with participants adhering to healthier lifestyles generally presenting at earlier disease stages. Because baseline disease severity may influence subsequent progression risk, this imbalance may partially affect the estimated associations. However, staged-adjustment sensitivity analyses that further adjusted for baseline CKM stage produced highly consistent results, suggesting that the observed associations were unlikely to be fully explained by baseline disease severity. Finally, all analyses in this study were conducted using data from the United Kingdom Biobank, whose participants are predominantly of European ancestry. Therefore, the generalizability of these findings to other ethnic populations or geographic settings may be limited. Future studies in independent cohorts with more diverse populations are needed to validate and extend these findings.

## 5 Conclusion

In conclusion, this large-scale cohort study suggests that adherence to a healthy lifestyle is associated with slower progression across CKM stages from early stages (0–3) to clinical CVD stages with or without renal failure, and ultimately to death. Our findings provide epidemiological evidence that may inform prevention strategies targeting CKM syndrome across disease stages, with lifestyle priorities potentially shifting as the disease progresses from primary to secondary prevention. Among the lifestyle factors examined, never smoking showed the strongest association with lower transition risks, highlighting its potential importance in CKM prevention. This may be complemented by multidimensional lifestyle strategies involving diet, physical activity, and alcohol control. Future research is needed to further clarify the causal relationships and biological mechanisms linking lifestyle factors to CKM syndrome, which may help guide the development of more precise and sustainable prevention strategies.

## Data Availability

The data analyzed in this study is subject to the following licenses/restrictions: The data utilized in this study are available from the United Kingdom Biobank upon approved application. Requests to access these datasets should be directed to: https://www.ukbiobank.ac.uk/.
