# Supplementary material for: Relative contributions of lifestyle factors to stage-specific progression and mortality in cardiovascular-kidney-metabolic syndrome: a prospective cohort study with multi-state models
Source: Front Public Health. 2026 Mar 20;14:1782700. doi: 10.3389/fpubh.2026.1782700 (PMC13047162; doi:10.3389/fpubh.2026.1782700)
Supplement: Supplementary file 1 [file Data_Sheet_1.pdf]

Relative contributions of lifestyle factors to stage-specific progression and mortality in Cardiovascular-Kidney-Metabolic syndrome: a prospective cohort study with multi-state models

Contents

Method S1. Details of the multi-state model 2
Method S2. Details of the quantile G-computation 4
Table S1. Methods for evaluating CKM stages 0-3 5
Table S2. International Classification of Disease used to ascertain incident outcomes 7
Table S3. Definitions and measurements of lifestyle factors in the UK Biobank study 8
Table S4. Test of normality 9
Table S5. Associations between lifestyle and risks of transitions in pattern A using multi-state model (model 1) 10
Table S6. Associations between lifestyle score and risks of transitions in pattern B using multi-state model (model 1) 11
Table S7. Results of subgroup analysis stratified by age (model 1) 12
Table S8. Results of subgroup analysis stratified by age (model 2) 13
Table S9. Results of subgroup analysis stratified by sex (model 1) 14
Table S10. Results of subgroup analysis stratified by sex (model 2) 15
Table S11. Association of lifestyle score with the disease transition pattern B, excluding outcome events that occurred in the first 2 years of follow-up 16
Table S12. Association of lifestyle score with disease transition pattern B, using a 1-day interval when entering different states on the same date 16
Table S13. Association of lifestyle score with disease transition pattern B, using a 3-day interval when entering different states on the same date 17
Table S14. Association of lifestyle score with disease transition pattern B, using a 5-day interval when entering different states on the same date 17
Table S15. Association of lifestyle score with disease transition pattern B, excluding participants entering different states on the same date 18
Table S16. Association of lifestyle score with disease transition pattern B, additionally adjusted for baseline CKM stage severity 18
Table S17. Association of lifestyle score with disease transition pattern B, additionally adjusted for BMI, systolic blood pressure, LDL cholesterol, and fasting blood glucose 19
Table S18. Baseline characteristics of participants included in the analysis and those excluded during cohort selection 20
Figure S1. Numbers (percentages) of participants in CKM transition pattern A by lifestyle score 21
Figure S2. Relative contributions of lifestyle factors to transition pattern A (model 1) 22
Figure S3. Relative contributions of lifestyle factors to transition pattern A (model 2) 23
Figure S4. The role of lifestyle factors in transition pattern B of CKM syndrome (model 1) 24
Figure S5. Relative contributions of lifestyle factors to transition pattern B (model 1) 25

Method S1. Details of the multi-state model

To characterize the longitudinal progression of CKM syndrome during follow-up and to simultaneously address multiple time-ordered and competing outcomes, this study employed multi-state models (MSMs). MSMs conceptualize the disease progression process as a continuous-time stochastic process, during which participants can transition between a finite set of mutually exclusive discrete states during follow-up. A movement between states is defined as a "transition" (i.e., an event occurrence). States can be classified as "transient" (allowing subsequent transitions) or "absorbing" (where transitions terminate, e.g., death), with specific definitions depending on the clinical or research context (e.g., specific symptoms, biomarkers, disease stages). Unlike traditional two-state survival models, which only capture whether a specific outcome occurs, multi-state models can explicitly model intermediate stages and terminal outcomes of a disease, thereby providing a more comprehensive picture of the dynamic disease trajectory and stage-specific risks.

Under the continuous-time multi-state modeling framework, the risk of transition between different states is quantitatively described by the transition intensity function (or instantaneous hazard function), defined as:

$$q_{ij}(t) = \lim_{\Delta t \to 0} \frac{Pr\{X(t+\Delta t) = j \mid X(t) = i\}}{\Delta t} \text{ , } i \neq j$$

where, for any two distinct states i and j, $X(t)$ denotes the state occupied by an individual at time t, and $q_{ij}(t)$ represents the instantaneous risk of transitioning from state i to state j at time t, given that the individual is still in state i. To adjust for covariates, covariates were expanded and specified separately for different transitions. According to the stages of CKM syndrome and the study objectives, the progression of CKM syndrome was modeled as an irreversible disease progression process, with death defined as the absorbing state, after which no further transitions occur.

In the preliminary analysis, we first established a multi-state model for the progression from the preclinical CKM phase to overall stage 4 and all-cause death, designated as Transition Pattern A. Below is the multi-state transition matrix for this pattern, comprising three disease states and three transition pathways: ① Baseline → CKM Stage 4; ② Baseline → Death; ③ CKM Stage 4 → Death.

From \ To	Baseline	CKM Stage 4	Death
Baseline	NA	1	2
CKM Stage 4	NA	NA	3
Death	NA	NA	NA

NA indicates that the transition between the two states does not exist; numbers 1, 2, and 3 indicate the pathway number.

Building on the preliminary findings, we further refined the classification of CKM Stage 4 and developed a new multi-state transition model as the primary analysis. Transition Pattern B includes four disease states and six transition pathways: ① Baseline → CKM Stage 4a; ② Baseline → CKM Stage 4b; ③ Baseline → Death; ④ CKM Stage 4a → CKM Stage 4b; ⑤ CKM Stage 4a → Death; ⑥ CKM Stage 4b → Death. The state transition matrix for Transition Pattern B is shown as follows.

From \ To	Baseline	CKM Stage 4a	CKM Stage 4b	Death
Baseline	NA	1	2	3
CKM Stage 4a	NA	NA	4	5
CKM Stage 4b	NA	NA	NA	6
Death	NA	NA	NA	NA

NA indicates that the transition between the two states does not exist; numbers 1 through 6 indicate the pathway number.

MSM analyses were performed using the mstate package in R software. All results are presented as hazard ratios (HRs) with 95% confidence intervals (CIs), used to quantify the strength of the association between exposure factors and the risk of transitioning along specific state transition pathways. Furthermore, for participants who entered different states on the same date, the theoretical entry date for the prior state was calculated as the entry date for the subsequent state minus 0.5 days to handle tied transition times.

Method S2. Details of the quantile G-computation

We used quantile G-computation (QGC) to estimate the relative contribution of a single lifestyle factor. The QGC obtains causal relationships and estimates positive or negative relative contribution for each component, which has been widely used in epidemiological research. In our study, the QGC method was carried out through the following steps:

Step 1: Arrangement of component data. The component data could keep the original scale, or be converted into categorized coded data as required, such as in quartiles. For the convenience of explanation, we directly used scores (0 or 1) for each lifestyle factor in the current study.

Step 2: Fitting regression models. The required covariates could be included in the model, which are omitted here for simplicity of notation. The model was as follows:

$$Y = \beta_0 + \sum_{j=1}^{k} \beta_j X_j^q + \epsilon$$

In the current study, k represented the total number of lifestyle components, and ϵ represented the residual term. x_j^q was the score of the jth lifestyle component. $\sum_j^k \beta_j$ was the mixture effect of the total lifestyles. Weight of component or relative contribution was defined as $\beta_j / \sum_j^k \beta_j$. It could be interpreted as the contribution of the jth component to the total effect when all the lifestyle components change from unhealthy to healthy at the same time. Given the overall lifestyle effect the weights are considered fixed and so do not have confidence intervals or p-values12. Because the estimation process of the above model did not limit the positive or negative of β_j, we could estimate the positive or negative weight of each component at the same time. When there were both positive and negative associations between components and outcome, the positive and negative weights were calculated separately, with all positive weights summing to 1 and all negative weights summing to 1.

Table S1. Methods for evaluating CKM stages 0-3

CKM stages	Threshold for CKM conditions
Stage 0: No CKM risk factors	All criteria are met：<br>① BMI <25 kg/m^2 (or <23 kg/m^2 if Asian ancestry)<br>② Waist circumference <88/102 cm in women/men (or if Asian ancestry <80/90 cm in women/men)<br>③ Fasting blood glucose <100 mg/dL and HbA1c <5.7% without self-reported diagnosis of diabetes or inclusion of "insulin" field in drug utilization reports<br>④ SBP <130 mm Hg and DBP <80 mm Hg without self-reported diagnosis of hypertension or inclusion of "Blood pressure medication" field in drug utilization reports<br>⑤ TG <135 mg/dL<br>⑥ eGFR ≥60 ml/min/1.73m^2 and without self-reported diagnosis of CKD<br>⑦ No self-reported diagnosis or inpatient diagnosis of clinical CVD<br>⑧ Framingham risk score <20
Stage 1: Excess or dysfunctional adiposity	Any of the three criteria is met:<br>① BMI ≥25 kg/m^2 (or ≥23 kg/m^2 if Asian ancestry)<br>② Waist circumference ≥88/102 cm in women/men (or if Asian ancestry ≥80/90 cm in women/men)<br>③ Fasting blood glucose ≥100–124 mg/dL and HbA1c ≥5.7%–6.4%<br>All criteria are met：<br>① SBP <130 mm Hg and DBP <80 mm Hg without self-reported diagnosis of hypertension or inclusion of "Blood pressure medication" field in drug utilization reports<br>② TG <135 mg/dL<br>③ eGFR ≥60 ml/min/1.73m2 and without self-reported diagnosis of CKD<br>④ No self-reported diagnosis or inpatient diagnosis of clinical CVD<br>⑤ Framingham risk score <20
Stage 2: Metabolic risk factors and CKD	Any of the five criteria is met:<br>① Fasting blood glucose ≥125 mg/dL and HbA1c ≥6.5% or self-reported diagnosis of diabetes or inclusion of "insulin" field in drug utilization reports<br>② SBP ≥130 mm Hg or DBP ≥80 mm Hg or self-reported diagnosis of hypertension or inclusion of "Blood pressure medication" field in drug utilization reports<br>③ metabolic syndrome*

	④ Hypertriglyceridemia (TG ≥135 mg/dL)<br>⑤ eGFR ≥30–60 ml/min/1.73m^2 and/or with self-reported diagnosis of CKD<br>All criteria are met：<br>① No self-reported diagnosis or inpatient diagnosis of clinical CVD<br>② Framingham risk score <20
Stage 3: Subclinical CVD in CKM	Any of the two criteria is met (on the basis of stages 0–2):<br>① eGFR <30 ml/min/1.73m^2<br>② Framingham risk score ≥20

* metabolic syndrome is defined by the presence of 3 or more of the following: (1) waist circumference ≥ 88 cm for women and ≥ 102 cm for men (≥ 80 cm for women and ≥ 90 cm for men if Asian ancestry); (2) HDL cholesterol < 40 mg/dL for men and < 50 mg/dL for women; (3) triglycerides ≥ 150 mg/dL; (4) elevated blood pressure (systolic blood pressure ≥ 130 mm Hg or diastolic blood pressure ≥ 80 mm Hg and/or use of antihypertensive medications); and (5) fasting blood glucose ≥ 100 mg/dL

CKM, cardiovascular-kidney-metabolic; CKD, chronic kidney disease; CVD, cardiovascular disease; eGFR, estimated glomerular filtration rate; TG, triglycerides; BMI, body mass index; SBP, systolic blood pressure; DBP, diastolic blood pressure; HbA1c, hemoglobin A1c; HDL, high-density lipoprotein

Table S2. International Classification of Disease used to ascertain incident outcomes

Variables	Subvariables	ICD-10 codes	UK Biobank field code
cardiovascular disease	coronary heart disease	I20, I20.0, I20.1, I20.8, I20.9, I21, I21.0, I21.1, I21.2, I21.3, I21.4, I21.9, I22, I22.0, I22.1, I22.8, I22.9, I23, I23.0, I23.1, I23.2, I23.3, I23.4, I23.5, I23.6, I23.8, I24, I24.0, I24.1, I24.8, I24.9, I25, I25.0, I25.1, I25.2, I25.5, I25.6, I25.8, I25.9	41202, 41262, 41270, 41280
	arrhythmias	I48, I49, I49.0, I49.1, I49.2, I49.3, I49.4, I49.5, I49.8, I49.9	
	heart failure	I11.0, I50, I50.0, I50.1, I50.9	
	stroke	I60, I60.0, I60.1, I60.2, I60.3, I60.4, I60.5, I60.6, I60.7, I60.8, I60.9, I61, I61.0, I61.1, I61.2, I61.3, I61.4, I61.5, I61.6, I61.8, I61.9, I63, I63.0, I63.1, I63.2, I63.3, I63.4, I63.5, I63.6, I63.8, I63.9, I64	
	peripheral artery disease	I70, I70.0, I70.00, I70.01, I70.2, I70.20, I70.21, I70.8, I70.80, I70.9, I70.90, I73.8, I73.9	
kidney failure	acute renal failure	N17, N17.0, N17.1, N17.2, N17.8, N17.9	
	chronic/ unspecified renal failure	N18.0, N18.5, N19	

ICD, International Classification of Disease

Table S3. Definitions and measurements of lifestyle factors in the UK Biobank study

Lifestyle factor	Details	Definition	UK Biobank field code
Smoking	Self-reported smoking status was categorized as never, former, or current. According to UK national guidelines, including those from the NHS and the National Institute for Health and Care Excellence, smoking cessation is strongly recommended. Participants who reported never smoking were classified under the healthy lifestyle category.	Never smoking was scored as 1.	20116
Alcohol consumption	Followed the NHS guidelines, which recommend that regardless of gender, alcohol consumption should not exceed 14 units per week.<br>1 unit of alcohol is defined as containing 8g of pure alcohol.<br>① Fortified wine (glass) = 1 unit<br>② Red wine (glass) = 1.5 units<br>③ White wine (glass) = 1.5 units<br>④ Beer (pint) = 2 units<br>⑤ Spirits (standard measure) = 1 unit	Moderate alcohol consumption was defined as ≤14 units/week,<br>Moderate alcohol consumption was scored as 1.	20117, 1558, 1568, 1578, 1588, 1598, 1608, 4407, 4418, 4429, 4440, 4451
Diet	Followed the DGA, low-risk diet was defined as an adequate intake of at least four of seven food groups recommended as dietary priorities.<br>① Fruits: ≥ 3 servings/day<br>② Vegetables: ≥ 3 servings/day<br>③ Fish: ≥ 2 servings/week<br>④ Processed meats: ≤ 1 serving/week<br>⑤ Unprocessed red meats: ≤ 1.5 servings/week<br>⑥ Whole grains: ≥ 3 servings/day<br>⑦ Refined grains: ≤1.5 servings/day	A healthy dietary pattern was defined as including at least four out of seven foods.<br>A healthy dietary pattern was scored as 1.	1309, 1319, 1289, 1299, 1329, 1339, 1349, 1369, 1379, 1389, 1438, 1448, 1458, 1468
Physical activity	According to World Health Organization guidelines on physical activity recommendation, adults should do ≥150 minutes of moderate activity per week	Meeting the criteria for healthy physical activity was scored as 1.	884, 894, 904, 914

	OR ≥ 75 minutes of vigorous activity per week OR an equivalent combination.		
Sleep	According to NHS recommendation, sleep duration of 7 to 9 hours was classified into the healthy lifestyle category.	Meeting the standard of healthy sleep is defined as 1.	1160
Sedentary behavior	According to WHO guidelines on sedentary behavior recommendations, sedentary behavior was measured by the sum of self-reported sedentary behavior hours spent watching TV and using a computer (do not include using a computer at work) during a typical day. Values greater than 24 hours per day were excluded.	Sedentary for less than 4 hours was scored as 1.	1070, 1080

NHS, National Health Service; DGA, Dietary Guidelines for Americans; WHO, World Health Organization

Table S4. Test of normality

Kolmogorov-Smirnov	***P*-value**
Age	<0.0001
Townsend deprivation index	<0.0001

Table S5. Associations between lifestyle and risks of transitions in pattern A using multi-state model (model 1)

	**Baseline → CKM stage 4**		**Baseline → Death**		**CKM stage 4 → Death**	
	HR (95%CI)	***P***	***HR (95%CI)***	***P***	***HR (95%CI)***	***P***
**Lifestyle score**						
[0~1]	1	··	1	··	1	··
[2~4]	0.68 (0.65, 0.70)	<0.001	0.61 (0.59, 0.64)	<0.001	0.66 (0.62, 0.70)	<0.001
[5~6]	0.49 (0.47, 0.52)	<0.001	0.45 (0.43, 0.48)	<0.001	0.46 (0.42, 0.51)	<0.001
Per score point	0.85 (0.84, 0.85)	<0.001	0.83 (0.82, 0.84)	<0.001	0.84 (0.82, 0.85)	<0.001
PAR (%)	36.85 (36.53, 37.19)	··	37.34 (37.03, 37.68)	··	47.24 (46.49, 48.09)	··
**lifestyle factors**						
Low-risk alcohol consumption	0.87 (0.85, 0.89)	<0.001	0.85 (0.83, 0.88)	<0.001	0.87 (0.82, 0.92)	<0.001
Low-risk diet	0.89 (0.87, 0.91)	<0.001	0.87 (0.84, 0.89)	<0.001	0.85 (0.81, 0.90)	<0.001
Never smoking	0.76 (0.74, 0.77)	<0.001	0.70 (0.68, 0.73)	<0.001	0.66 (0.63, 0.70)	<0.001
Low-risk sleep	0.82 (0.80, 0.85)	<0.001	0.86 (0.83, 0.89)	<0.001	0.93 (0.88, 0.98)	0.009
Regular physical activity	0.89 (0.87, 0.91)	<0.001	0.85 (0.83, 0.88)	<0.001	0.85 (0.81, 0.90)	<0.001
Low-to-moderate sedentary behavior	0.85 (0.83, 0.87)	<0.001	0.88 (0.86, 0.91)	<0.001	0.91 (0.87, 0.96)	0.001

HR, hazard ratio; CI, confidence interval; ··, not applicable (reference group or statistic not calculated).
Model was adjusted for age and sex.

Table S6. Associations between lifestyle score and risks of transitions in pattern B using multi-state model (model 1)

Transitions	Low [0~1]		Medium [2~4]		High [5~6]		Per score point		PAR (%)
	HR (95%CI)	*P*	*HR (95%CI)*	*P*	*HR (95%CI)*	*P*	*HR (95%CI)*	*P*	
Baseline → CKM stage 4a	1	··	0.70 (0.67, 0.72)	<0.001	0.52 (0.50, 0.55)	<0.001	0.86 (0.85, 0.86)	<0.001	33.58 (33.27, 33.90)
Baseline → CKM stage 4b	1	··	0.51 (0.46, 0.57)	<0.001	0.28 (0.25, 0.33)	<0.001	0.74 (0.71, 0.76)	<0.001	83.31 (82.71, 83.87)
Baseline → Death	1	··	0.61 (0.59, 0.64)	<0.001	0.45 (0.43, 0.48)	<0.001	0.83 (0.82, 0.84)	<0.001	37.34 (37.03, 37.68)
CKM stage 4a → CKM stage 4b	1	··	0.70 (0.63, 0.77)	<0.001	0.43 (0.37, 0.50)	<0.001	0.84 (0.82, 0.87)	<0.001	62.97 (62.20, 63.87)
CKM stage 4a → Death	1	··	0.66 (0.60, 0.71)	<0.001	0.46 (0.40, 0.53)	<0.001	0.84 (0.82, 0.86)	<0.001	46.50 (45.69, 47.44)
CKM stage 4b → Death	1	··	0.85 (0.77, 0.94)	0.001	0.86 (0.73, 1.01)	0.061	0.96 (0.93, 0.99)	0.006	1.97 (1.44, 2.57)

HR, hazard ratio; CI, confidence interval; ··, not applicable (reference group).
Model was adjusted for age and sex.

Table S7. Results of subgroup analysis stratified by age (model 1)

**Transitions**	**Lifestyle score**	***HR* (95% *CI*)**		***P* for interaction**
		**Young (<60)**	**Old (≥60)**	
Baseline → CKM stage 4a	[0~1]	1	1	··
	[2~4]	0.63 (0.60, 0.67)	0.74 (0.71, 0.77)	<0.001
	[5~6]	0.43 (0.39, 0.46)	0.58 (0.55, 0.62)	<0.001
	Per score point	0.82 (0.81, 0.83)	0.88 (0.87, 0.89)	<0.001
Baseline → CKM stage 4b	[0~1]	1	1	··
	[2~4]	0.46 (0.38, 0.55)	0.54 (0.48, 0.61)	0.301
	[5~6]	0.21 (0.15, 0.28)	0.32 (0.27, 0.38)	0.021
	Per score point	0.68 (0.65, 0.72)	0.76 (0.73, 0.78)	0.004
Baseline → Death	[0~1]	1	1	··
	[2~4]	0.54 (0.50, 0.58)	0.65 (0.62, 0.69)	<0.001
	[5~6]	0.38 (0.34, 0.42)	0.50 (0.46, 0.54)	<0.001
	Per score point	0.80 (0.78, 0.81)	0.85 (0.84, 0.86)	<0.001
CKM stage 4a → CKM stage 4b	[0~1]	1	1	··
	[2~4]	0.59 (0.49, 0.71)	0.74 (0.66, 0.83)	0.001
	[5~6]	0.31 (0.21, 0.44)	0.47 (0.40, 0.57)	0.016
	Per score point	0.79 (0.75, 0.84)	0.86 (0.83, 0.88)	<0.001
CKM stage 4a → Death	[0~1]	1	1	··
	[2~4]	0.57 (0.48, 0.68)	0.68 (0.62, 0.75)	0.007
	[5~6]	0.43 (0.32, 0.58)	0.47 (0.41, 0.55)	0.589
	Per score point	0.79 (0.75, 0.83)	0.85 (0.83, 0.88)	<0.001
CKM stage 4b → Death	[0~1]	1	1	··
	[2~4]	0.77 (0.64, 0.94)	0.87 (0.78, 0.97)	0.238
	[5~6]	0.75 (0.52, 1.07)	0.89 (0.74, 1.06)	0.601
	Per score point	0.92 (0.87, 0.99)	0.97 (0.93, 1.00)	0.095

HR, hazard ratio; CI, confidence interval; ··, not applicable (reference group).
Model 1 was adjusted for age and sex.

Table S8. Results of subgroup analysis stratified by age (model 2)

Transitions	Lifestyle score	*HR* (95% *CI*)		*P* for interaction
		Young (<60)	Old (≥60)	
Baseline → CKM stage 4a	[0~1]	1	1	··
	[2~4]	0.69 (0.65, 0.73)	0.77 (0.74, 0.80)	<0.001
	[5~6]	0.51 (0.47, 0.55)	0.63 (0.59, 0.67)	<0.001
	Per score point	0.85 (0.84, 0.86)	0.89 (0.88, 0.90)	<0.001
Baseline → CKM stage 4b	[0~1]	1	1	··
	[2~4]	0.54 (0.45, 0.65)	0.59 (0.52, 0.66)	0.420
	[5~6]	0.29 (0.21, 0.39)	0.38 (0.32, 0.45)	0.040
	Per score point	0.73 (0.69, 0.78)	0.78 (0.76, 0.81)	0.012
Baseline → Death	[0~1]	1	1	··
	[2~4]	0.59 (0.55, 0.64)	0.69 (0.65, 0.73)	<0.001
	[5~6]	0.44 (0.40, 0.48)	0.54 (0.50, 0.58)	<0.001
	Per score point	0.82 (0.81, 0.84)	0.87 (0.85, 0.88)	<0.001
CKM stage 4a → CKM stage 4b	[0~1]	1	1	··
	[2~4]	0.63 (0.52, 0.76)	0.79 (0.70, 0.89)	0.001
	[5~6]	0.36 (0.25, 0.51)	0.53 (0.44, 0.64)	0.018
	Per score point	0.82 (0.77, 0.87)	0.88 (0.85, 0.91)	<0.001
CKM stage 4a → Death	[0~1]	1	1	··
	[2~4]	0.65 (0.55, 0.78)	0.72 (0.65, 0.80)	0.007
	[5~6]	0.54 (0.40, 0.72)	0.52 (0.44, 0.60)	0.617
	Per score point	0.83 (0.78, 0.88)	0.87 (0.85, 0.90)	0.001
CKM stage 4b → Death	[0~1]	1	1	··
	[2~4]	0.77 (0.63, 0.95)	0.88 (0.78, 0.99)	0.209
	[5~6]	0.74 (0.51, 1.07)	0.89 (0.74, 1.07)	0.611
	Per score point	0.93 (0.87, 0.99)	0.97 (0.93, 1.00)	0.107

HR, hazard ratio; CI, confidence interval; ··, not applicable (reference group).

Model 2 was adjusted for age, sex, ethnic background, TDI, education, and assessment center.

Table S9. Results of subgroup analysis stratified by sex (model 1)

Transitions	Lifestyle score	*HR* (95% *CI*)		*P* for interaction
		Male	Female	
Baseline → CKM stage 4a	[0~1]	1	1	··
	[2~4]	0.74 (0.71, 0.77)	0.61 (0.58, 0.65)	<0.001
	[5~6]	0.57 (0.54, 0.61)	0.45 (0.41, 0.48)	<0.001
	Per score point	0.87 (0.86, 0.89)	0.83 (0.82, 0.84)	<0.001
Baseline → CKM stage 4b	[0~1]	1	1	··
	[2~4]	0.59 (0.52, 0.67)	0.39 (0.33, 0.46)	<0.001
	[5~6]	0.32 (0.26, 0.39)	0.22 (0.18, 0.28)	0.023
	Per score point	0.76 (0.73, 0.79)	0.71 (0.67, 0.74)	0.017
Baseline → Death	[0~1]	1	1	··
	[2~4]	0.62 (0.59, 0.66)	0.60 (0.56, 0.64)	0.426
	[5~6]	0.45 (0.42, 0.49)	0.45 (0.41, 0.49)	0.918
	Per score point	0.83 (0.81, 0.84)	0.84 (0.82, 0.85)	0.249
CKM stage 4a → CKM stage 4b	[0~1]	1	1	··
	[2~4]	0.77 (0.68, 0.86)	0.55 (0.46, 0.65)	0.002
	[5~6]	0.44 (0.36, 0.54)	0.37 (0.29, 0.48)	0.323
	Per score point	0.86 (0.83, 0.89)	0.81 (0.78, 0.86)	0.105
CKM stage 4a → Death	[0~1]	1	1	··
	[2~4]	0.71 (0.64, 0.79)	0.55 (0.47, 0.64)	0.004
	[5~6]	0.44 (0.37, 0.53)	0.45 (0.36, 0.54)	0.954
	Per score point	0.84 (0.81, 0.87)	0.84 (0.81, 0.88)	0.970
CKM stage 4b → Death	[0~1]	1	1	··
	[2~4]	0.84 (0.75, 0.95)	0.86 (0.72, 1.02)	0.910
	[5~6]	0.93 (0.75, 1.15)	0.81 (0.64, 1.03)	0.345
	Per score point	0.96 (0.92, 1.00)	0.96 (0.91, 1.00)	0.782

HR, hazard ratio; CI, confidence interval; ··, not applicable (reference group).
Model 1 was adjusted for age and sex.

Table S10. Results of subgroup analysis stratified by sex (model 2)

Transitions	Lifestyle score	*HR* (95% *CI*)		*P* for interaction
		Male	Female	
Baseline → CKM stage 4a	[0~1]	1	1	··
	[2~4]	0.77 (0.74, 0.81)	0.66 (0.62, 0.71)	<0.001
	[5~6]	0.63 (0.59, 0.67)	0.51 (0.48, 0.55)	<0.001
	Per score point	0.89 (0.88, 0.90)	0.86 (0.84, 0.87)	<0.001
Baseline → CKM stage 4b	[0~1]	1	1	··
	[2~4]	0.65 (0.57, 0.73)	0.45 (0.38, 0.53)	<0.001
	[5~6]	0.38 (0.31, 0.47)	0.29 (0.23, 0.36)	0.047
	Per score point	0.79 (0.76, 0.82)	0.74 (0.71, 0.78)	0.033
Baseline → Death	[0~1]	1	1	··
	[2~4]	0.67 (0.63, 0.71)	0.63 (0.58, 0.68)	0.689
	[5~6]	0.51 (0.47, 0.56)	0.49 (0.44, 0.53)	0.751
	Per score point	0.85 (0.84, 0.87)	0.85 (0.84, 0.87)	0.147
CKM stage 4a → CKM stage 4b	[0~1]	1	1	··
	[2~4]	0.83 (0.73, 0.93)	0.57 (0.48, 0.69)	0.005
	[5~6]	0.50 (0.40, 0.62)	0.41 (0.31, 0.52)	0.519
	Per score point	0.88 (0.85, 0.91)	0.83 (0.78, 0.87)	0.190
CKM stage 4a → Death	[0~1]	1	1	··
	[2~4]	0.77 (0.69, 0.85)	0.59 (0.51, 0.69)	0.012
	[5~6]	0.50 (0.41, 0.60)	0.50 (0.41, 0.62)	0.661
	Per score point	0.86 (0.84, 0.89)	0.86 (0.83, 0.90)	0.720
CKM stage 4b → Death	[0~1]	1	1	··
	[2~4]	0.84 (0.74, 0.95)	0.86 (0.72, 1.02)	0.709
	[5~6]	0.93 (0.74, 1.15)	0.79 (0.62, 1.02)	0.447
	Per score point	0.96 (0.92, 1.00)	0.95 (0.90, 1.00)	0.861

HR, hazard ratio; CI, confidence interval; ··, not applicable (reference group).

Model 2 was adjusted for age, sex, ethnic background, TDI, education, and assessment center.

Table S11. Association of lifestyle score with the disease transition pattern B, excluding outcome events that occurred in the first 2 years of follow-up

Transitions	Low [0~1]		Medium [2~4]		High [5~6]		Per score point	
	HR (95%CI)	*P*	*HR (95%CI)*	*P*	*HR (95%CI)*	*P*	*HR (95%CI)*	*P*
Baseline → CKM stage 4a	1	··	0.74 (0.72, 0.77)	<0.001	0.59 (0.56, 0.62)	<0.001	0.88 (0.87, 0.89)	<0.001
Baseline → CKM stage 4b	1	··	0.58 (0.52, 0.64)	<0.001	0.35 (0.30, 0.41)	<0.001	0.77 (0.75, 0.79)	<0.001
Baseline → Death	1	··	0.66 (0.63, 0.69)	<0.001	0.51 (0.48, 0.54)	<0.001	0.86 (0.84, 0.87)	<0.001
CKM stage 4a → CKM stage 4b	1	··	0.74 (0.67, 0.83)	<0.001	0.52 (0.44, 0.61)	<0.001	0.87 (0.84, 0.90)	<0.001
CKM stage 4a → Death	1	··	0.73 (0.66, 0.80)	<0.001	0.53 (0.46, 0.61)	<0.001	0.87 (0.84, 0.89)	<0.001
CKM stage 4b → Death	1	··	0.86 (0.77, 0.96)	0.006	0.86 (0.73, 1.03)	0.096	0.96 (0.93, 0.99)	0.017

HR, hazard ratio; CI, confidence interval; ··, not applicable (reference group).
Model was adjusted for age, sex, ethnic background, TDI, education, and assessment center.

Table S12. Association of lifestyle score with disease transition pattern B, using a 1-day interval when entering different states on the same date

Transitions	Low [0~1]		Medium [2~4]		High [5~6]		Per score point	
	HR (95%CI)	*P*	*HR (95%CI)*	*P*	*HR (95%CI)*	*P*	*HR (95%CI)*	*P*
Baseline → CKM stage 4a	1	··	0.74 (0.71, 0.77)	<0.001	0.58 (0.56, 0.61)	<0.001	0.88 (0.87, 0.89)	<0.001
Baseline → CKM stage 4b	1	··	0.57 (0.52, 0.63)	<0.001	0.35 (0.30, 0.41)	<0.001	0.77 (0.75, 0.79)	<0.001
Baseline → Death	1	··	0.65 (0.62, 0.68)	<0.001	0.50 (0.47, 0.53)	<0.001	0.85 (0.84, 0.86)	<0.001
CKM stage 4a → CKM stage 4b	1	··	0.75 (0.68, 0.82)	<0.001	0.49 (0.42, 0.57)	<0.001	0.86 (0.84, 0.89)	<0.001
CKM stage 4a → Death	1	··	0.71 (0.65, 0.77)	<0.001	0.52 (0.46, 0.60)	<0.001	0.86 (0.84, 0.89)	<0.001
CKM stage 4b → Death	1	··	0.85 (0.77, 0.94)	0.002	0.86 (0.73, 1.01)	0.066	0.96 (0.93, 0.99)	0.008

HR, hazard ratio; CI, confidence interval; ··, not applicable (reference group).
Model was adjusted for age, sex, ethnic background, TDI, education, and assessment center.

Table S13. Association of lifestyle score with disease transition pattern B, using a 3-day interval when entering different states on the same date

Transitions	Low [0~1]		Medium [2~4]		High [5~6]		Per score point	
	HR (95%CI)	*P*	*HR (95%CI)*	*P*	*HR (95%CI)*	*P*	*HR (95%CI)*	*P*
Baseline → CKM stage 4a	1	··	0.74 (0.71, 0.77)	<0.001	0.58 (0.56, 0.61)	<0.001	0.88 (0.87, 0.89)	<0.001
Baseline → CKM stage 4b	1	··	0.57 (0.52, 0.63)	<0.001	0.35 (0.30, 0.41)	<0.001	0.77 (0.75, 0.79)	<0.001
Baseline → Death	1	··	0.65 (0.62, 0.68)	<0.001	0.50 (0.47, 0.53)	<0.001	0.85 (0.84, 0.86)	<0.001
CKM stage 4a → CKM stage 4b	1	··	0.75 (0.68, 0.82)	<0.001	0.49 (0.42, 0.57)	<0.001	0.86 (0.84, 0.89)	<0.001
CKM stage 4a → Death	1	··	0.71 (0.65, 0.77)	<0.001	0.52 (0.46, 0.60)	<0.001	0.86 (0.84, 0.89)	<0.001
CKM stage 4b → Death	1	··	0.85 (0.77, 0.94)	0.002	0.86 (0.73, 1.01)	0.066	0.96 (0.93, 0.99)	0.008

HR, hazard ratio; CI, confidence interval; ··, not applicable (reference group).
Model was adjusted for age, sex, ethnic background, TDI, education, and assessment center.

Table S14. Association of lifestyle score with disease transition pattern B, using a 5-day interval when entering different states on the same date

Transitions	Low [0~1]		Medium [2~4]		High [5~6]		Per score point	
	HR (95%CI)	*P*	*HR (95%CI)*	*P*	*HR (95%CI)*	*P*	*HR (95%CI)*	*P*
Baseline → CKM stage 4a	1	··	0.74 (0.71, 0.77)	<0.001	0.58 (0.56, 0.61)	<0.001	0.88 (0.87, 0.89)	<0.001
Baseline → CKM stage 4b	1	··	0.57 (0.52, 0.63)	<0.001	0.35 (0.30, 0.41)	<0.001	0.77 (0.75, 0.79)	<0.001
Baseline → Death	1	··	0.65 (0.62, 0.68)	<0.001	0.50 (0.47, 0.53)	<0.001	0.85 (0.84, 0.86)	<0.001
CKM stage 4a → CKM stage 4b	1	··	0.75 (0.68, 0.82)	<0.001	0.49 (0.42, 0.57)	<0.001	0.86 (0.84, 0.89)	<0.001
CKM stage 4a → Death	1	··	0.71 (0.65, 0.77)	<0.001	0.52 (0.46, 0.60)	<0.001	0.86 (0.84, 0.89)	<0.001
CKM stage 4b → Death	1	··	0.85 (0.77, 0.94)	0.002	0.86 (0.73, 1.01)	0.066	0.96 (0.93, 0.99)	0.008

HR, hazard ratio; CI, confidence interval; ··, not applicable (reference group).
Model was adjusted for age, sex, ethnic background, TDI, education, and assessment center.

Table S15. Association of lifestyle score with disease transition pattern B, excluding participants entering different states on the same date

**Transitions**	**Low [0~1]**		**Medium [2~4]**		**High [5~6]**		**Per score point**	
	HR (95%CI)	***P***	***HR (95%CI)***	***P***	***HR (95%CI)***	***P***	***HR (95%CI)***	***P***
Baseline → CKM stage 4a	1	··	0.74 (0.71, 0.77)	<0.001	0.58 (0.56, 0.61)	<0.001	0.88 (0.87, 0.89)	<0.001
Baseline → CKM stage 4b	1	··	0.57 (0.52, 0.64)	<0.001	0.35 (0.30, 0.41)	<0.001	0.77 (0.75, 0.79)	<0.001
Baseline → Death	1	··	0.65 (0.62, 0.68)	<0.001	0.50 (0.47, 0.53)	<0.001	0.85 (0.84, 0.86)	<0.001
CKM stage 4a → CKM stage 4b	1	··	0.75 (0.68, 0.82)	<0.001	0.49 (0.42, 0.57)	<0.001	0.86 (0.84, 0.89)	<0.001
CKM stage 4a → Death	1	··	0.71 (0.65, 0.78)	<0.001	0.52 (0.46, 0.60)	<0.001	0.86 (0.84, 0.89)	<0.001
CKM stage 4b → Death	1	··	0.86 (0.77, 0.94)	0.002	0.86 (0.73, 1.01)	0.074	0.96 (0.93, 0.99)	0.007

HR, hazard ratio; CI, confidence interval; ··, not applicable (reference group).
Model was adjusted for age, sex, ethnic background, TDI, education, and assessment center.

Table S16. Association of lifestyle score with disease transition pattern B, additionally adjusted for baseline CKM stage severity

**Transitions**	**Low [0~1]**		**Medium [2~4]**		**High [5~6]**		**Per score point**	
	HR (95%CI)	***P***	***HR (95%CI)***	***P***	***HR (95%CI)***	***P***	***HR (95%CI)***	***P***
Baseline → CKM stage 4a	1	··	0.77(0.75,0.80)	<0.001	0.64(0.62,0.68)	<0.001	0.90(0.89,0.91)	<0.001
Baseline → CKM stage 4b	1	··	0.62(0.56,0.69)	<0.001	0.43(0.37,0.50)	<0.001	0.80(0.78,0.83)	<0.001
Baseline → Death	1	··	0.67(0.64,0.70)	<0.001	0.53(0.50,0.56)	<0.001	0.86(0.85,0.87)	<0.001
CKM stage 4a → CKM stage 4b	1	··	0.78(0.70,0.86)	<0.001	0.54(0.46,0.64)	<0.001	0.89(0.86,0.91)	<0.001
CKM stage 4a → Death	1	··	0.71(0.65,0.78)	<0.001	0.53(0.46,0.61)	<0.001	0.87(0.84,0.89)	<0.001
CKM stage 4b → Death	1	··	0.85(0.77,0.94)	0.001	0.84(0.71,0.99)	0.038	0.95(0.92,0.98)	0.003

HR, hazard ratio; CI, confidence interval; ··, not applicable (reference group).
Model was adjusted for age, sex, ethnic background, TDI, education, assessment center, and baseline CKM stage severity.

Table S17. Association of lifestyle score with disease transition pattern B, additionally adjusted for BMI, systolic blood pressure, LDL cholesterol, and fasting blood glucose

Transitions	Low [0~1]		Medium [2~4]		High [5~6]		Per score point	
	HR (95%CI)	*P*	*HR (95%CI)*	*P*	*HR (95%CI)*	*P*	*HR (95%CI)*	*P*
Baseline → CKM stage 4a	1	··	0.79(0.76,0.82)	<0.001	0.66(0.63,0.70)	<0.001	0.90(0.89,0.91)	<0.001
Baseline → CKM stage 4b	1	··	0.66(0.60,0.74)	<0.001	0.47(0.40,0.55)	<0.001	0.82(0.80,0.85)	<0.001
Baseline → Death	1	··	0.66(0.63,0.69)	<0.001	0.52(0.49,0.55)	<0.001	0.86(0.85,0.87)	<0.001
CKM stage 4a → CKM stage 4b	1	··	0.81(0.73,0.89)	<0.001	0.57(0.49,0.67)	<0.001	0.90(0.87,0.93)	<0.001
CKM stage 4a → Death	1	··	0.70(0.65,0.77)	<0.001	0.51(0.45,0.59)	<0.001	0.86(0.84,0.88)	<0.001
CKM stage 4b → Death	1	··	0.85(0.77,0.94)	0.001	0.84(0.71,0.99)	0.033	0.95(0.92,0.98)	0.003

HR, hazard ratio; CI, confidence interval; ··, not applicable (reference group).
Model was adjusted for age, sex, ethnic background, TDI, education, assessment center, BMI, systolic blood pressure, LDL cholesterol, and fasting blood glucose.

Table S18. Baseline characteristics of participants included in the analysis and those excluded during cohort selection

Variable	Included participants	Excluded participants
N	308657	193486
Age, year	57 (50, 63)	59 (51, 64)
Male (%)	138359 (44.83)	90623 (46.84)
White (%)	295446 (95.72)	176929 (92.34)
Education (%)		
No qualification	42489 (13.77)	42742 (23.07)
Any other qualifications	158500 (51.36)	88538 (47.79)
College or university degree	107668 (34.87)	53988 (29.14)
TDI	−2.30 (−3.71, 0.15)	-1.83 (-3.5, 1.19)
Lifestyle factors (%)		
Low-risk alcohol consumption	172096 (55.75)	114752 (59.31)
Low-risk diet	162115 (52.53)	63036 (47.37)
Never smoking	136265 (44.15)	90487 (47.27)
Low-risk sleep	77899 (25.23)	58704 (30.34)
Regular physical activity	127940 (41.45)	87335 (45.14)
Low-to-moderate sedentary behavior	146590 (47.49)	93706 (51.85)
CKM stages (%)		
0	27360 (8.87)	2627 (4.52)
1	21563 (6.99)	2749 (4.73)
2	163266 (52.89)	28982 (49.87)
3	96468 (31.25)	23752 (40.87)

Values are presented as median (Q1, Q3) or n (%).

Percentages were calculated based on the number of participants with non-missing data for each variable.

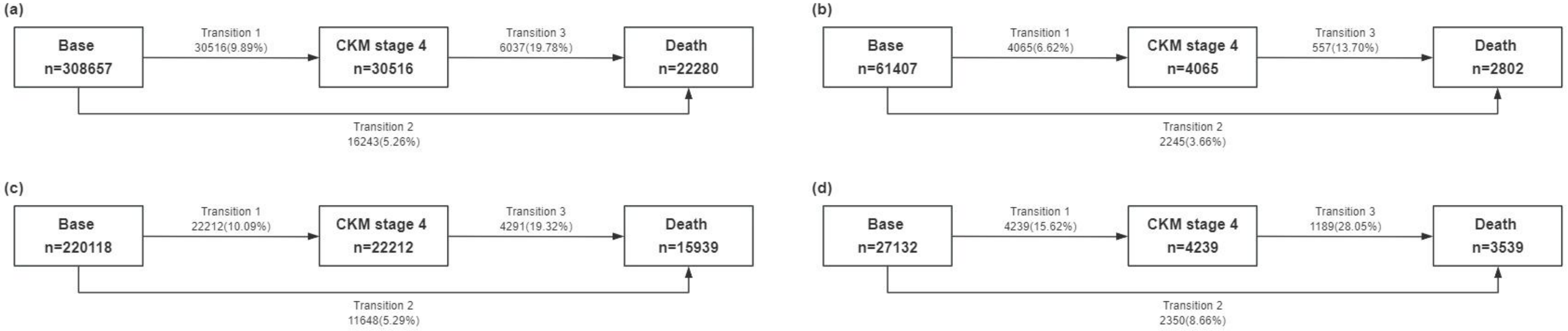

Figure S1. Numbers (percentages) of participants in CKM transition pattern A by lifestyle score

(a) Overall population; (b) Lifestyle score 5–6 group; (c) Lifestyle score 2–4 group; (d) Lifestyle score 0–1 group.

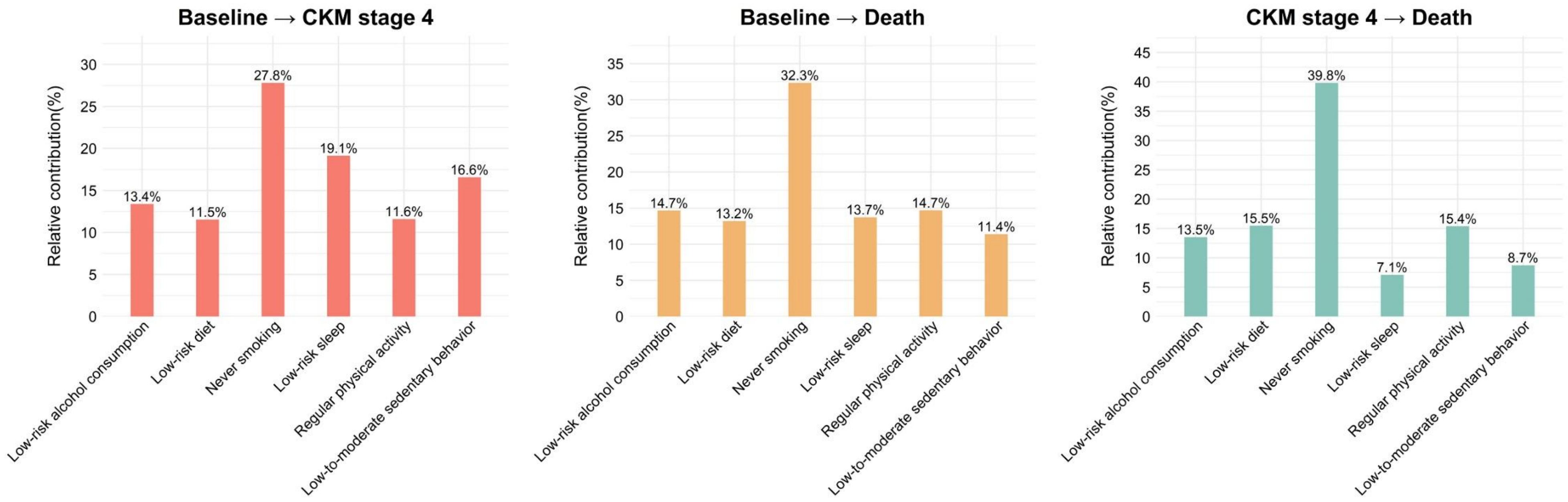

Figure S2. Relative contributions of lifestyle factors to transition pattern A (model 1)

Relative contribution estimations of lifestyle factors with the disease dynamic progression were using quantile G-computation. Model was adjusted for age and sex.

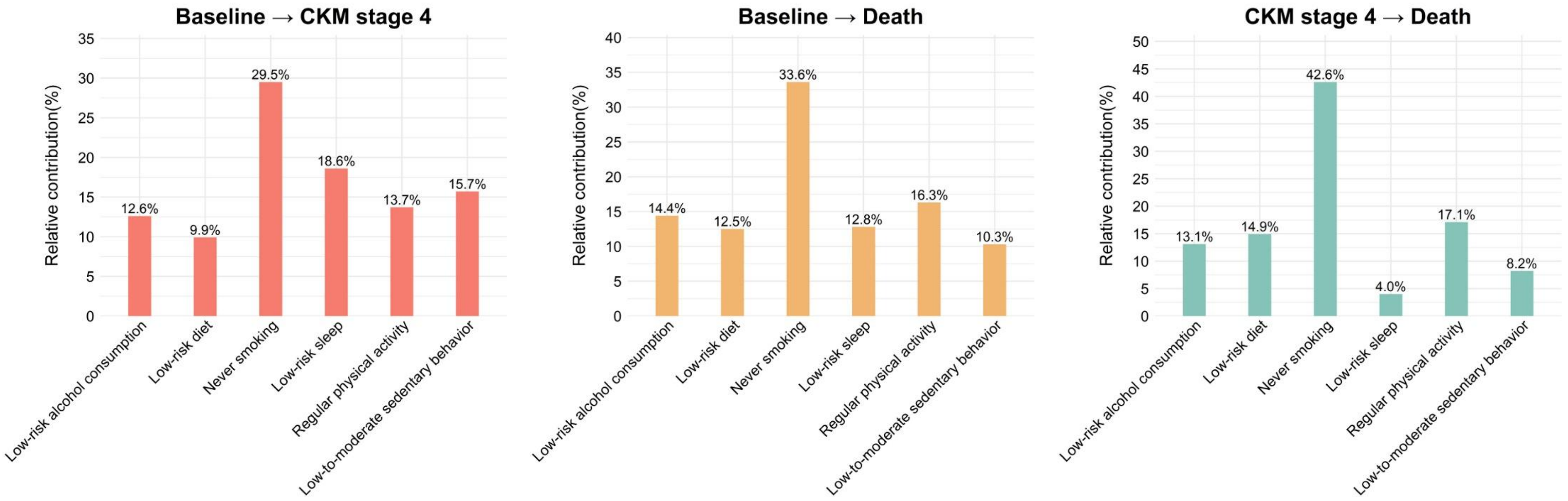

Figure S3. Relative contributions of lifestyle factors to transition pattern A (model 2)

Relative contribution estimations of lifestyle factors with the disease dynamic progression were using quantile G-computation. Model was adjusted for age, sex, ethnic background, TDI, education, and assessment center.

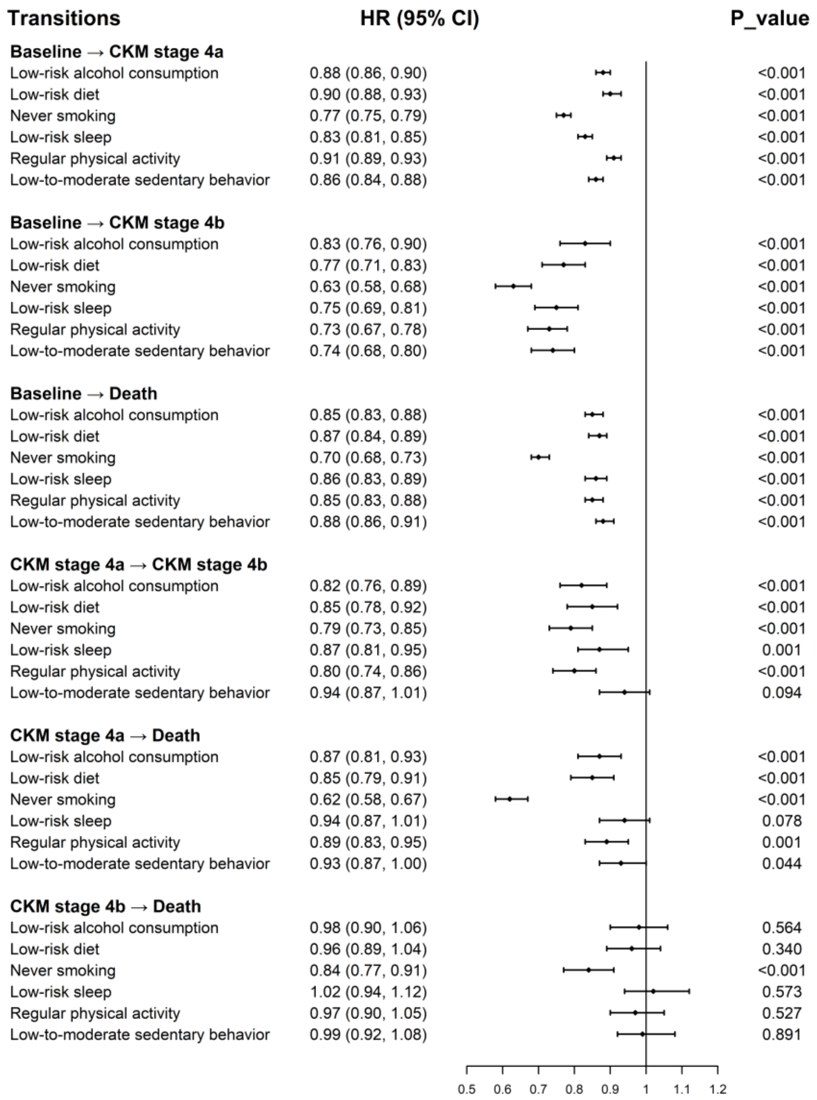

Figure S4. The role of lifestyle factors in transition pattern B of CKM syndrome (model 1)

HR, hazard ratio; CI, confidence interval. Model was adjusted for age and sex.

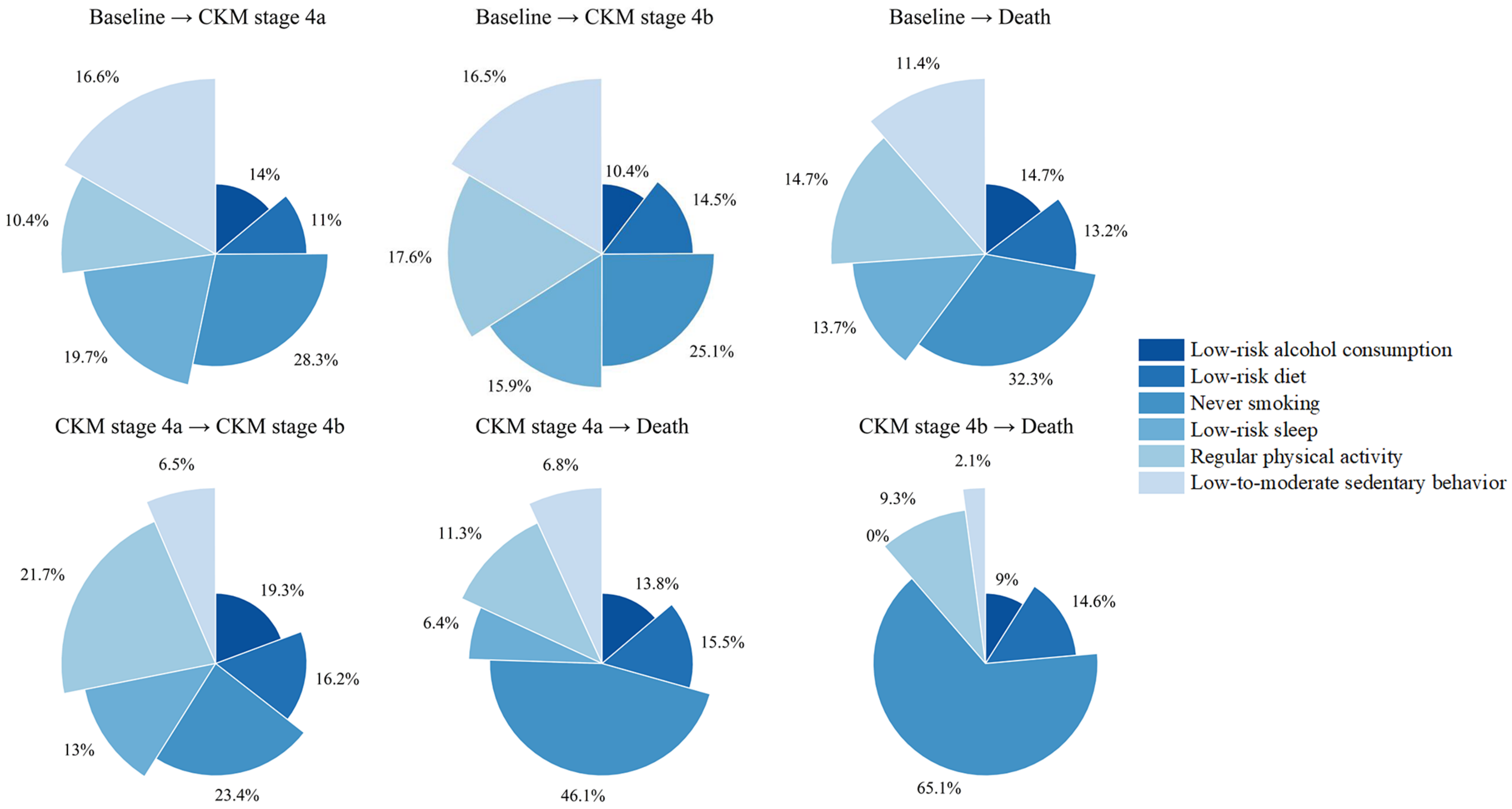

Figure S5. Relative contributions of lifestyle factors to transition pattern B (model 1)

Quantile G-computation was used to quantify the relative contributions of lifestyle factors to CKM progression. The bars illustrate the contributions of each factor. Model was adjusted for age and sex.
